# Supplementary material for: DECRYPT trial: study protocol for a phase II randomised controlled trial of cognitive therapy for post-traumatic stress disorder (PTSD) in youth exposed to multiple traumatic stressors
Source: BMJ Open. 2021 Jul 1;11(7):e047600. doi: 10.1136/bmjopen-2020-047600 (PMC8252885; doi:10.1136/bmjopen-2020-047600)
Supplement: Author's manuscript [file bmjopen-2020-047600.draft_revisions.pdf]

BMJ Open is committed to open peer review. As part of this commitment we make the peer review history of every article we publish publicly available.

When an article is published we post the peer reviewers' comments and the authors' responses online. We also post the versions of the paper that were used during peer review. These are the versions that the peer review comments apply to.

The versions of the paper that follow are the versions that were submitted during the peer review process. They are not the versions of record or the final published versions. They should not be cited or distributed as the published version of this manuscript.

BMJ Open is an open access journal and the full, final, typeset and author-corrected version of record of the manuscript is available on our site with no access controls, subscription charges or pay-per-view fees (http://bmjopen.bmj.com).

If you have any questions on BMJ Open's open peer review process please email info.bmjopen@bmj.com

BMJ Open

The DECRYPT trial: Study protocol for a phase II randomised controlled trial of cognitive therapy for post-traumatic stress disorder (PTSD) in youth exposed to multiple traumatic stressors.

Journal:	*BMJ Open*
Manuscript ID	bmjopen-2020-047600
Article Type:	Protocol
Date Submitted by the Author:	03-Dec-2020
Complete List of Authors:	Allen, Leila; University of East Anglia, Department of Clinical Psychology and Psychological Therapies, Norwich Medical School<br>Ashford, Polly-Anna; University of East Anglia, Norwich Clinical Trials Unit, Norwich Medical School<br>Beeson, Ella; Hertfordshire Partnership University NHS Foundation Trust<br>Byford, Sarah; King's College London, Centre for the Economics of Mental and Physical Health<br>Chow, Jessica; University of East Anglia, Department of Clinical Psychology and Psychological Therapies, Norwich Medical School<br>Dalgleish, Tim; MRC Cognition and Brain Sciences Unit; Cambridgeshire and Peterborough NHS Foundation Trust<br>Danese, Andrea; King's College London Institute of Psychiatry Psychology and Neuroscience, Department of Child and Adolescent Psychiatry; King's College London, Social, Genetic and Developmental Psychiatry Centre<br>Finn, Jack; University of East Anglia, Department of Clinical Psychology and Psychological Therapies, Norwich Medical School<br>Goodall, Ben; North East London NHS Foundation Trust<br>Grainger, Lauren; University of East Anglia, Department of Clinical Psychology and Psychological Therapies, Norwich Medical School<br>Hammond, Matthew ; University of East Anglia, Norwich Clinical Trials Unit, Norwich Medical School<br>Humphrey, Ayla; Cambridgeshire and Peterborough NHS Foundation Trust<br>Mahoney-Davies, Gerwyn; Cardiff and Vale University Health Board<br>Morant, Nicola; University College London,<br>Shepstone, Lee; University of East Anglia, Norwich Clinical Trials Unit, Norwich Medical School<br>Sims, Erika; University of East Anglia, Norwich Clinical Trials Unit, Norwich Medical School<br>Smith, Patrick; King's College London Institute of Psychiatry Psychology and Neuroscience, Department of Psychology<br>Stallard, Paul; University of Bath, Department for Health<br>Swanepoel, Annie; Hertfordshire Partnership University NHS Foundation Trust<br>Trickey, David ; Anna Freud Centre<br>Trigg, Katie; University of East Anglia, Department of Clinical Psychology and Psychological Therapies, Norwich Medical School

	Wilson, Jon; Norfolk and Suffolk NHS Foundation Trust,<br>Meiser-Stedman , Richard ; University of East Anglia, Department of Clinical Psychology and Psychological Therapies

Keywords:	Child & adolescent psychiatry < PSYCHIATRY, Anxiety disorders < PSYCHIATRY, Depression & mood disorders < PSYCHIATRY

SCHOLARONE™
Manuscripts

I, the Submitting Author has the right to grant and does grant on behalf of all authors of the Work (as defined in the below author licence), an exclusive licence and/or a non-exclusive licence for contributions from authors who are: i) UK Crown employees; ii) where BMJ has agreed a CC-BY licence shall apply, and/or iii) in accordance with the terms applicable for US Federal Government officers or employees acting as part of their official duties; on a worldwide, perpetual, irrevocable, royalty-free basis to BMJ Publishing Group Ltd ("BMJ") its licensees and where the relevant Journal is co-owned by BMJ to the co-owners of the Journal, to publish the Work in this journal and any other BMJ products and to exploit all rights, as set out in our licence.

The Submitting Author accepts and understands that any supply made under these terms is made by BMJ to the Submitting Author unless you are acting as an employee on behalf of your employer or a postgraduate student of an affiliated institution which is paying any applicable article publishing charge ("APC") for Open Access articles. Where the Submitting Author wishes to make the Work available on an Open Access basis (and intends to pay the relevant APC), the terms of reuse of such Open Access shall be governed by a Creative Commons licence – details of these licences and which Creative Commons licence will apply to this Work are set out in our licence referred to above.

Other than as permitted in any relevant BMJ Author's Self Archiving Policies, I confirm this Work has not been accepted for publication elsewhere, is not being considered for publication elsewhere and does not duplicate material already published. I confirm all authors consent to publication of this Work and authorise the granting of this licence.

For peer review only

The DECRYPT trial: Study protocol for a phase II randomised controlled trial of cognitive therapy for post-traumatic stress disorder (PTSD) in youth exposed to multiple traumatic stressors.

Leila Allen[1], Polly-Anna Ashford[2], Ella Beeson[3], Sarah Byford[4], Jessica Chow[1], Tim Dalgleish[5,6], Andrea Danese[7,8], Jack Finn[1], Ben Goodall, Lauren Grainger[1], Matt Hammond[2], Ayla Humphrey[6], Gerwyn Mahoney-Davies[9], Nicola Morant[10], Lee Shepstone[2], Erika Sims[2], Patrick Smith[12], Paul Stallard[13], Annie Swanepoel[3]; David Trickey[14]; Katie Trigg[1], Jon Wilson[15], Richard Meiser-Stedman[1]

[1] Department of Clinical Psychology & Psychological Therapies, Norwich Medical School, University of East Anglia

[2] Norwich Clinical Trials Unit, Norwich Medical School, University of East Anglia

[3] Hertfordshire Partnership Foundation NHS Trust

[4] King's Health Economics, King's College London, London

[5] Medical Research Council Cognition and Brain Sciences Unit, University of Cambridge

[6] Cambridgeshire and Peterborough NHS Foundation Trust

[7] Social, Genetic and Developmental Psychiatry Centre, King's College London

[8] Department of Child and Adolescent Psychiatry, Institute of Psychiatry, Psychology and Neuroscience, King's College London

[9] North East London NHS Foundation Trust

[10] Cardiff & Vale University Health Board

[11] Division of Psychiatry, Faculty of Brain Sciences, University College London

For peer review only - http://bmjopen.bmj.com/site/about/guidelines.xhtml

[12] Department of Psychology, Institute of Psychiatry, Psychology and Neuroscience, King's College London

[13] Department for Health, University of Bath

[14] Anna Freud National Centre for Children and Families, London

[15] Norfolk & Suffolk Foundation NHS Trust

Corresponding author:

Richard Meiser-Stedman, Department of Clinical Psychology & Psychological Therapies, Norwich Medical School, University of East Anglia, email: r.meiser-stedman@uea.ac.uk

For peer review only

For peer review only - http://bmjopen.bmj.com/site/about/guidelines.xhtml

Abstract

Background: Post-traumatic stress disorder (PTSD) is a distressing and disabling condition that affects significant numbers of children and adolescents. Youth exposed to multiple traumas (e.g. abuse, domestic violence) are at particular risk of developing PTSD. Cognitive-Therapy for PTSD (CT-PTSD), derived from adult work, is a theoretically-informed, disorder-specific form of trauma-focused cognitive-behavioural therapy (TF-CBT). While efficacious for child and adolescent single-event trauma samples, its effectiveness in routine settings with more complex, multiple-trauma exposed youth has not been established. The Delivery of Cognitive Therapy for Young People after Trauma (DECRYPT) randomised controlled trial (RCT) examines the effectiveness of CT-PTSD for treating PTSD following multiple trauma exposure in children and young people in comparison to treatment as usual (TAU).

Methods/Design: This protocol describes a two-arm, patient-level, single blind, superiority RCT comparing CT-PTSD (n=60) to TAU (n=60) in children and young people aged 8-17 years with a diagnosis of PTSD following multiple trauma exposure. The primary outcome is PTSD severity assessed using the Children's Revised Impact of Event Scale (8-item version) at post-treatment (i.e. approximately five months post-randomisation). Secondary outcomes include structured interview assessment for PTSD, complex PTSD symptoms, depression and anxiety, overall functioning and parent-rated mental health. Mid-treatment and 11- and 29-month post-randomisation assessments will also be completed. Process-outcome evaluation will consider which mechanisms underpin or moderate recovery. Qualitative interviews with the young people, their families and their therapists will be undertaken. Cost-effectiveness of CT-PTSD relative to TAU will be also be assessed.

Ethics and dissemination: This trial protocol has been approved by a UK Health Research Authority (HRA) Research Ethics Committee (East of England – Cambridge South, 16/EE/0233). Findings will be disseminated broadly via peer-reviewed empirical journal articles, conference presentations, and clinical workshops.

For peer review only - http://bmjopen.bmj.com/site/about/guidelines.xhtml

Trial registration: ISRCTN, ISRCTN12077707. Registered 24th October 2016, http://www.isrctn.com/ISRCTN12077707

For peer review only

For peer review only - http://bmjopen.bmj.com/site/about/guidelines.xhtml

Strengths and limitations of this study

- This study will involve a highly pragmatic evaluation of a psychological therapy (Cognitive Therapy for post-traumatic stress disorder [PTSD]) for children and young people with PTSD, being embedded in front-line UK National Health Service child and adolescent mental health services (CAMHS).
- The trial will include youth with the more severe, multiple trauma histories typically seen in CAMHS, and in addition to questionnaire and interview measures of PTSD, the trial will consider Complex PTSD symptoms.
- The trial will explore putative mediators and moderators of treatment outcome, and also includes a cost-effectiveness evaluation and qualitative data collection.
- There is no standard "treatment as usual" for this population, and it is anticipated that treatment in this control arm will vary.

For peer review only

For peer review only - http://bmjopen.bmj.com/site/about/guidelines.xhtml

Administrative information

Scientific title: Cognitive Therapy for the treatment of post-traumatic stress disorder (PTSD) in youth exposed to multiple traumatic stressors: a phase II randomised controlled trial

Trial registration: ISRCTN12077707

Keywords: Post traumatic stress disorder, Children, Young people, PTSD, Cognitive therapy, multiple trauma.

Trial status: Open to recruitment (Opened 1st March 2017; anticipated recruitment end date, 31st May 2021)

Protocol version: 4.0 (15th April 2020)

Word count: 4876

For peer review only

For peer review only - http://bmjopen.bmj.com/site/about/guidelines.xhtml

Introduction

Background

Posttraumatic stress disorder (PTSD) is a deeply distressing and disabling psychiatric disorder. PTSD in youth is usually comorbid with other psychiatric conditions,[1 2] and may persist for years or even decades if untreated [3 4]. The UK Royal College of Psychiatrists estimates[5] the prevalence of PTSD in UK youth to be 3%, while a recent epidemiological study suggested that over 7% of UK youth will have developed PTSD at some point by the age of 18.[6]

Traumatic events, defined[7] as those that involve exposure to actual or threatened death, serious injury, or sexual violence, are experienced by up to two-thirds of children by age 16.[8 9] A significant minority of trauma-exposed children and adolescents go on to develop posttraumatic stress disorder (PTSD).[1 10] For those young people subjected to *multiple* traumatic stressors (e.g. physical or sexual abuse, witnessing domestic violence, war or community violence), the clinical presentation of PTSD is particularly severe.[11 12] The life-long impact of multiple trauma exposure in childhood is well documented, with dramatically increased prevalence of a range of emotional disorders, compromised educational, occupational and social functioning, increased use of mental health services and poor physical health.[13-18] PTSD has been shown to have a uniquely harmful role in determining the long-term response to childhood trauma exposure, by mediating the relationship between such experiences and a range of physical and mental health outcomes later in childhood[19 20] and adulthood[21-23], including psychosis.[24]

In adults, empirical support for trauma-focused cognitive behaviour therapy (TF-CBT) is substantial.[25-27] TF-CBT typically comprises several core elements: psychoeducation, exposure (to help desensitise patients to trauma memories), cognitive elements (to reframe the meanings and interpretations associated with trauma and its aftermath), and coping management (e.g. problem-solving, anxiety management). Over the past 15 years several randomised controlled trials have been conducted that support the efficacy of TF-CBT (using a variety of specific manuals) in children and

For peer review only - http://bmjopen.bmj.com/site/about/guidelines.xhtml

adolescents.[28] While this evidence shows considerable promise, a number of important issues remain to be resolved.

First, existing trials rarely compare an experimental treatment to treatment as usual, i.e. active treatment/clinical care from a child and adolescent mental health service. Moreover, many studies have not focused on cases of the kind typically referred to mental health services, instead focusing on youth identified in child protection/social services settings. Similarly, many trials have not used "front-line therapists", reducing the ability to implement trial findings in routine settings.

Second, the cost-effectiveness of implementing a trauma-focused psychological intervention (such as different forms of TF-CBT) in youth mental health services has received little consideration. Evaluating the health economic implications of the investment of time in training and supervising therapists in delivering such specific treatments is essential for the future service delivery.

Third, it is important to consider whether a cognitive therapy for PTSD (CT-PTSD) treatment protocol, a particular form of TF-CBT, can be utilised in child and adolescent mental health services by front-line therapists. Step increases in treatment efficacy for adults with PTSD have been achieved through careful individual formulation and the enhanced use of theoretically-derived techniques in CT-PTSD.[39-41] The cognitive model of PTSD has been largely supported in children and youth.[29] While CT-PTSD adapted for youth[30 31] has been shown to be efficacious for PTSD following single event trauma when delivered in research clinics[31 32], its effectiveness in routine clinical settings with youth exposed to multiple traumas has yet to be established.

Fourth, the responsiveness of Complex PTSD symptoms in children and adolescents to psychological treatment is still poorly understood. Symptoms of Complex PTSD include the defining criteria of PTSD alongside disruptions in emotion regulation, relational capacities and self-concept, and are associated with multiple trauma exposures (e.g. sexual or physical abuse). While the understanding of Complex PTSD in adults has seen significant advances over recent years (with the disorder now included in ICD-11), little is known about the nature and presentation of Complex

For peer review only - http://bmjopen.bmj.com/site/about/guidelines.xhtml

PTSD in youth, and our treatments for these youth lag behind provision for adults. The ability of youth with high levels of Complex PTSD symptoms to derive benefit from psychological therapies for PTSD is largely unknown. However, the first study to address this issue in youth found that Complex PTSD symptoms were responsive to TF-CBT, and that the presence of Complex PTSD did not lessen the efficacy of TF-CBT to treat PTSD symptoms.[33]

Fifth, moderators and mediators of treatment responsiveness are still poorly understood. While change in negative trauma-related misappraisals has been shown to underpin treatment response for cognitive-behavioural therapies[31 32], other potential mechanisms have received scant or no attention. In particular, potential contraindications for cognitive-behavioural therapies for PTSD in youth have received comparatively little attention. Shedding light on these factors would inform decision making about treatments for youth with PTSD and clarify the essential "ingredients" of successful treatment.

Sixth, the lived experiences of youth undergoing treatment for PTSD following multiple traumas has received little consideration. Qualitative methods may offer important insights into the acceptability and feasibility of delivering treatments like CT-PTSD for youth with PTSD. Such insights may direct how treatments are evaluated and delivered in the future, as well as informing clinician attitudes to the management of this population's needs.

Objectives

This study aims to fill these gaps in the literature by addressing several objectives. The primary objective of the study is to evaluate whether CT-PTSD is an effective treatment for PTSD symptoms in 8-17 year old youth who have been exposed to multiple traumatic stressors, relative to TAU in UK National Health Service (NHS) child and adolescent/youth mental health services. The secondary objectives of the study are as follows: (1) is CT-PTSD effective in the treatment of complex PTSD, anxiety, depression, general functioning and parent-related mental health in youth with PTSD, relative to TAU? (2) Is the treatment cost-effective, relative to TAU? (3) If effective

For peer review only - http://bmjopen.bmj.com/site/about/guidelines.xhtml

relative to TAU, by what mechanisms does CT-PTSD have its effect? (4) What demographic, symptomatic, cognitive and psychosocial factors moderate response to treatment? (5) What are the views and experiences of youth with PTSD, their parents, and therapists, about receiving or delivering CT-PTSD, and how do these inform judgements of the acceptability and feasibility of CT-PTSD?

Given the significant uncertainties over the effect size associated with both CT-PTSD and TAU in this context and the ability of therapists in settings to deliver CT-PTSD with this relatively complex population, this study was considered to be a Phase II trial. It is anticipated that the trial will inform the development of a later definitive trial of CT-PTSD for this population.

Study design

This study is a two-arm, patient-level, single blind, superiority RCT comparing CT-PTSD to treatment-as-usual (TAU). Participants will be allocated to CT-PTSD or TAU according to a 1:1 ratio, with stratification by baseline Child Revised Impact of Event Scale (8-item version; CRIES-8) score and recruiting site. The CRIES-8 is the routine outcome monitoring measure for PTSD in UK child and adolescent mental health services (CAMHS).

This protocol has been written in accordance with the Standard Protocol Items: Recommendations for Interventional Trials (SPIRIT) 2013 statement.[34]

Methods: Participants, interventions, and outcomes

Study setting

Trial data collection, randomisation, blinding and data analysis will be overseen by the Norwich Clinical Trials Unit (NCTU). Participants will be recruited from NHS CAMHS and youth mental health services in England and Wales.

Eligibility criteria

For peer review only - http://bmjopen.bmj.com/site/about/guidelines.xhtml

A total of 120 children/young people aged 8-17 years will be randomised to either CT-PTSD (n=60) or TAU (n=60). Youth are eligible to be included in the study if they: (1) meet the criteria for a diagnosis of PTSD, as defined by the Diagnostic and Statistical Manual of Mental Disorders, Fifth Edition (DSM-5);[7] (2) score 17 or greater on the CRIES-8; and (3) have been exposed to multiple traumatic stressors.

Exclusion criteria are: (1) *change* of prescribed psychiatric medication within the past two months (though receiving medication was not an exclusion criterion); (2) pervasive developmental disorder or neurodevelopmental disorder (e.g. autism, but not ADHD); (3) intellectual disability; (4) another primary psychiatric diagnosis or clinical need that warrants treatment ahead of PTSD (e.g. psychosis, suicidal behaviour, conduct disorder); (5) inability to speak English; (6) on-going exposure to threat (e.g. living with an abuser; regularly placing self in danger) or safeguarding issues; (7) strong likelihood of being unable to complete treatment (e.g. imminent house or foster placement move); or (8) history of organic brain damage.

Interventions

CT-PTSD. CT-PTSD for PTSD is a structured, fully manualised [30] psychological treatment delivered in an individual format for children and adolescents. The proposed frequency and duration of treatment is up to 15 treatment sessions (typically 10-12, of 60-90 minutes duration).

CT-PTSD involves several core elements: psychoeducation, with an emphasis on the role of cognitive processes in the onset and maintenance of PTSD; narrative work and imaginal reliving to help develop a coherent trauma narrative; cognitive restructuring (to reframe the meanings and interpretations associated with trauma and its aftermath), and coping management (e.g. addressing maladaptive strategies such as thought suppression, rumination and safety-seeking behaviours). Up to three sessions will be allowed for stabilising other comorbid conditions and difficulties (e.g. depression or self-harm).

For peer review only - http://bmjopen.bmj.com/site/about/guidelines.xhtml

CT-PTSD will be delivered by NHS CAMHS/youth mental health service therapists who will have completed training in CT-PTSD by a member of the trial team. Trial therapists who deliver CT-PTSD must have an appropriate professional qualification (e.g. as a nurse, occupational therapist, clinical psychologist, social worker, psychiatrist or BABCP-registered cognitive-behavioural therapist) and be approved by their local site Principal Investigator to act as a trial therapist.

CT-PTSD will be delivered wherever is permitted and feasible for local therapists (e.g. in NHS mental health clinics, local GP surgeries, at home). Supervision will be provided by a clinical psychologist from the trial team. Following the completion of a course of CT-PTSD, usual NHS care arrangements will apply for participants in this arm.

TAU. Mental health professionals and others involved in the care of the participants in the TAU arm will be encouraged to provide whatever help they deem necessary, e.g. general clinical management, supportive counselling, family therapy, medication. TAU must involve an active treatment, i.e. it cannot involve being on a waiting list. Since TF-CBT has been a recommended treatment for PTSD in youth in the UK since 2005,[25] no therapists in the TAU arm were prevented from delivering this intervention; they were not, however, trained by the research team to deliver the CT-PTSD intervention. Therapist contact in the TAU arm would not be prescribed by trial participation in any way, with one exception: the participants will receive no contact with the trained trial therapists delivering CT-PTSD, and their therapist/clinician will not receive supervision for that case from a trained trial therapist.

Treatment integrity

Trial therapists will be supervised by members of the trial team while working with trial participants. Trial therapists delivering CT-PTSD will record sessions with participants and rate the use of specific treatment techniques in notes and recording sheets. Trial collaborators will oversee the quality assurance of the training and will monitor and ensure treatment adherence.

Outcomes

For peer review only - http://bmjopen.bmj.com/site/about/guidelines.xhtml

The timing of all measures and assessments is presented in Figure 1.

Primary clinical outcome. The primary outcome measure is PTSD severity using the Child Revised Impact of Events Scale (CRIES-8) score at post-treatment (approximately five months post-randomisation). The CRIES-8, a validated self-report questionnaire,[35] is the routine outcome monitoring tool for PTSD in children and adolescents endorsed by the Children and Young People's programme – Improving Access to Psychological Therapies (CYP-IAPT). The CRIES-8 will also be completed at baseline, 2.5 months post-randomisation (mid-treatment), and 11 months and 29 months post-randomisation.

Secondary clinical outcomes. PTSD diagnosis and symptoms using the Child PTSD Symptom Scale for DSM-5, interviewer version (CPSS-I-5; structured interview, psychometric properties made available by authors),[36] with additional items for measuring dissociation and complex PTSD. The Child and Adolescent Trauma Screen (CATS) will also be used as a self-report questionnaire to address all DSM-5 PTSD symptoms,[37] with additional items measuring dissociation and complex PTSD. Anxiety and depression will be assessed using the Revised Child Anxiety and Depression Scale (RCADS).[38] Suicidal ideation will be assessed using five items from the Mood and Feelings Questionnaire (MFQ).[39] Affect regulation and irritability will be assessed by the Affective Reactivity Index (ARI; child and parent/caregiver-report versions).[40] Clinician-rated general functioning will be assessed using the Children's Global Assessment Scale (CGAS).[41] Parent/caregiver-rated mental health and well-being will be indexed by the Strengths and Difficulties Questionnaire (SDQ).[42] Parent/caregiver-rated borderline personality traits will be assessed using the McLean Screening Instrument for Borderline Personality Disorder, caregiver version (MSI-BPD-C).[43]

In order to ascertain whether parent/caregiver mental health moderates their children's ability to benefit from intervention, and whether parent/caregiver mental health improve with their child receiving intervention, clinical outcomes for parents/caregivers will be assessed. Parent/caregiver

For peer review only - http://bmjopen.bmj.com/site/about/guidelines.xhtml

depression, anxiety and post-traumatic stress will be assessed using the Patient Health Questionnaire (PHQ-9),[44] Generalised Anxiety Disorder Assessment (GAD-7),[45] and Posttraumatic Stress Diagnostic Scale, DSM-5 version (PDS-5),[46] respectively.

Health economic evaluation measures. Resource use will be collected using the Child and Adolescent Service Use Schedule (CA-SUS),[47] modified for a PTSD population, a structured economic interview for youth mental health populations. The CA-SUS will be administered by trial team members at baseline and trained assessors (blinded to allocation) at post-treatment and 11 month follow up assessments. The interview involves both the young person (if aged 12 or above) and the young person's primary parent/caregiver. Effectiveness will be measured using the EQ-5D-Y[48] measure of health-related quality of life, a preference-based, generic measure capable of generating quality adjusted life years (QALYs).

Process measures. Items from an existing interview[49] will be used to assess whether a young person is experiencing hearing voices. Social support will be assessed using the Multidimensional Scale of Perceived Social Support.[50] Several mechanisms proposed by cognitive theories for PTSD will be measured: trauma-related appraisals (the Children's Post-Traumatic Cognitions Inventory, CPTCI),[51] trauma memory quality (the Trauma Memory Quality Scale, TMQQ),[52] trauma-related rumination and self-blame[53] and safety-seeking behaviours (the Child Safety Behaviour Scale, CSBS).[54] Treatment credibility will be assessed using four items derived from a previous trial.[55] Therapeutic alliance will be assessed using the Therapeutic Alliance Scale for Children, revised (TASC-r; completed by participant).[56]

Qualitative data. Semi-structured interviews will be conducted to explore the perspectives of youth receiving CT-PTSD, family members, and therapists who deliver CT-PTSD. Interviews topic guides will be tailored to each of these perspectives, adapted to be age- appropriate for youth respondents, and reviewed by Patient and Public Involvement (PPI) representatives. They will explore experiences and perceived impacts of treatment and its constituent components; the acceptability and

For peer review only - http://bmjopen.bmj.com/site/about/guidelines.xhtml

feasibility of the treatment received / delivered; and views of being involved in a randomised controlled trial and the assessment processes involved. Interviews with youth will be conducted after their post-treatment assessment. In line with sample size guidelines for qualitative interviews,[57] sample sizes of 12-15 for youth and family member interviews are planned, sampled to include youth across the trial age spectrum. A similar sample size is planned for trial therapists.

Participant timeline

Participants will be assessed five times during the study: baseline, mid-treatment (approximately 2.5 months post-randomisation), post-treatment (approximately five months post-randomisation) and at 11- and 29-month post-randomisation follow up assessments (approximately 6- and 24-months post-treatment; see Figure 1).

Sample size

This is the first trial to compare CT-PTSD (or any other treatment) to routine care in NHS CAMHS and youth mental health settings for children and young people with PTSD following multiple trauma exposure. While two waiting-list controlled trials have been conducted of the CT-PTSD treatment package in research clinics,[31 32] no UK trials have evaluated the effectiveness of routine care for the treatment of PTSD in young people with multiple trauma exposure, or the effectiveness of CT-PTSD as delivered by therapists working in routine clinical settings. As such, no reliable estimates of effect size are available. An attempt was made to estimate likely effect size based on meta-analyses of existing psychological therapies for PTSD in children and adolescents. The waiting-list controlled effect size of CT-PTSD was greater than 1.2 in each of the studies to have examined this intervention to date.[31 32] The Cochrane meta-analysis of treatments for PTSD in children and adolescents[10] yielded an effect size of 1.05 for all psychological therapies versus control conditions, whereas for CBT versus control conditions, the effect size was 1.34. However, these effect sizes are likely to overestimate the efficacy of CBT; few studies were included in the headline analyses, and those that were included were waiting-list controlled, rather than using an active

For peer review only - http://bmjopen.bmj.com/site/about/guidelines.xhtml

control condition (e.g. TAU or other interventions that involved clinician/therapist contact). A high quality trial of another psychological therapy (prolonged exposure, a form of TF-CBT) for adolescent girls with PTSD following sexual abuse, that used supportive counselling as a control intervention, yielded a controlled (i.e. between groups) effect size of 1.0.[36] An alternative meta-analysis,[58] including a broader range of studies to evaluate CBT in youth with PTSD (many of which used active control treatments such as supportive counselling) suggested a controlled effect size of around 0.67; a more recent meta-analysis[59] suggested an effect size for TF-CBT of .79. The more conservative effect size estimate of .67 was used for the proposed trial.

In order to have 90% power to detect a between groups effect size of 0.67 (two-tailed t-test, 0.05 significance level) a sample size of 96 (48 participants per group) is required; a combined sample size of 96 would have 80% power to detect an effect size of .58 (two-tailed t-test, 0.05 significance level). In order to account for drop out (estimated at 20%), 120 participants will be recruited.

Recruitment

Participants will be recruited from NHS Trusts in England and Wales. Clinicians in participating teams will be encouraged to contact the trial team to discuss potential participants to ensure that they meet inclusion criteria. If a child or young person appears eligible, the clinician will share information with the participant and their family about the study who will be given at least 48 hours to decide whether they wish to participate. Consenting families will be contacted by the trial team to discuss further and to arrange baseline assessments; trial eligibility will be confirmed following this assessment. Children and young people will not be able to enter DECRYPT unless a therapist within the team is available to offer CT-PTSD and another clinician/therapist is available to offer TAU, i.e. TAU cannot involve a waiting list.

Methods: Assignment of interventions

For peer review only - http://bmjopen.bmj.com/site/about/guidelines.xhtml

Allocation

Following pre-trial assessments, consenting participants will be randomised to study arms stratified by CRIES-8 score (17-28, 29-40) and site (i.e. NHS Trust). An online randomisation service managed by NCTU will assign allocation to groups. Allocation is by pre-set lists of permuted blocks with randomly distributed block sizes (determined by the trial statistician). The lists will be generated by the Data Management Team at NCTU. The trial manager will enrol participants. Following randomisation, participants, their clinical team and their General Practitioner (GP) will be notified of their allocation by the trial manager. See Figure 2 below for the Consolidated Standards of Reporting Trials (CONSORT) diagram.

Blinding

Trained assessors collecting post-treatment and follow-up data will be blinded to group allocation. These assessments will be undertaken by trained assessors with no other role in the trial. Following allocation to CT-PTSD or TAU, all participants in the study, their care coordinator/ referrer and clinical team (if applicable) are asked not to reveal the group to which the participants were randomised to the trained assessor.

Methods: Data collection, management, and analysis

Data collection methods

Quantitative outcome variable data will be collected using paper forms and entered electronically on to the trial database, or online. Qualitative data will be collected using face-to-face interviews, or where this is not possible telephone or video-call interviews. An appropriately trained individual will undertake semi-structured interviews with participants and their families after treatment has been completed. For participants that have withdrawn from treatment or the trial but consent to taking part in a qualitative interview, this will be completed when appropriate. Interviews will be audio-recorded and transcribed verbatim, with all identifying information removed.

For peer review only

For peer review only - http://bmjopen.bmj.com/site/about/guidelines.xhtml

Data management

To maintain confidentiality, each participant will be given a unique trial Participant Identification Number (PIN). Data will be entered under this identification number onto the central database stored on the servers based at NCTU. The database and coding frames have been developed by the Clinical Trial Manager in conjunction with NCTU. The database software provides a number of features to help maintain data quality, including maintaining an audit trail, allowing custom validations on all data, allowing users to raise data query requests, and search facilities to identify validation failure/ missing data. After completion of the trial, the database will be retained on the servers of NCTU for on-going analysis of secondary outcomes.

Statistical methods

A full statistical analysis plan (SAP) will be written and agreed with the Data Monitoring Committee (DMC) and Trial Steering Committee (TSC) prior to database lock and any data analysis. This plan will be amended only with agreement of the former two committees.

Primary analyses will compare CT-PTSD with TAU on CRIES-8 scores at post-treatment. The primary analysis will be on the intention to treat principal: i.e. all participants will be followed up for data collection irrespective of adherence to treatment and will be analysed according to group allocation rather than intervention received. Assuming a normal distribution (potentially of transformed values), a linear model will be constructed. This will include recruiting site (as a random factor), CRIES-8 at baseline (as a covariate) and any factors considered prognostic and determined in advance of any analysis, together with treatment arm as a fixed effect.

The primary intention to treat analysis is intended to provide inferences regarding the effectiveness of the intervention overall not to provide inferences regarding the causal effect of the intervention itself, but on the intervention as deployed in 'real life'. Statistical significance will be set at the conventional (two-tailed) 5% level and all parameter estimates will be presented with 95% confidence intervals. Analyses will be carried out by the trial statistician blinded to group identity,

For peer review only - http://bmjopen.bmj.com/site/about/guidelines.xhtml

(i.e. 'subgroup' blind). There are no plans for formal interim efficacy or subgroup analyses. Analyses will be carried out in SAS (currently version 9.4).

Qualitative analysis

Qualitative data will be analysed using thematic analysis within NVivo software.[60] A collaborative approach throughout the analytic process in which emerging themes are discussed between those who collect data and other members of the research team will enhance reflexivity and validity.[61] The views and experiences of each group (children/young people, parents/caregivers, trial therapists) will be analysed both separately and in comparison to each other in order to gain a multi-perspective view of the acceptability and feasibility of delivering CT-PTSD in routine NHS child and youth mental health services. Both commonalities and variations within and between these stakeholder groups will be explored.

Health economics evaluation

Economic evaluation will compare CT-PTSD with TAU at the 11-month follow-up. It will take the NHS/personal social services perspective, preferred by NICE,[62] but will additionally include education services, given the age of the population. Service use data will be collected using the CA-SUS while health-related quality of life will be assessed using the EQ-5D-Y (see above). Data on CT-PTSD contacts and TAU contacts and on indirect time for the intervention (e.g. supervision, training) will be collected directly from therapists/clinicians and service records; participants will be asked not to mention any CAMHS/mental health contacts so as to reduce the possibility of unblinding and double-counting of service contacts. Service use estimates will be combined with standard UK sources for unit costs to estimate total costs (including NHS reference costs for hospital contacts, the British National Formulary for medications and the PSSRU Unit Costs of Health and Social Care). The cost of CT-PTSD will be directly calculated.[63]

Economic analysis will be carried out on an intention-to-treat basis using an analysis plan to be drawn up prior to data analysis. Costs and outcomes will be compared and presented in terms of

For peer review only - http://bmjopen.bmj.com/site/about/guidelines.xhtml

mean differences and 95% confidence intervals obtained by non-parametric bootstrap regression to account for the non-normal distribution commonly found in economic data.[64] Cost-effectiveness will be assessed through the calculation of incremental cost-effectiveness ratios (the additional cost of one intervention compared with another divided by the additional effects). The primary analysis will focus on effects measured using the CRIES-8, known to be sensitive to change in this population. Secondary analyses will explore cost-effectiveness in terms of QALYs derived from the EQ-5D-Y and using the area under the curve approach,[65] a more policy relevant measure that is preferred by NICE, but with unknown sensitivity in young people with PTSD. Uncertainty will be explored using cost-effectiveness planes and cost-effectiveness acceptability curves based on the net-benefit approach.[66 67] These curves are an alternative to confidence intervals around ICERs and show the probability that one intervention is cost-effective compared to the other, for a range of values that a decision maker would be willing to pay for an additional unit of an outcome. All economic analyses will be adjusted for CRIES-8 score and site, in line with the clinical analyses, plus baseline values of the variables of interest (cost, QALYs), to provide a more relevant treatment-effect estimate.[68]

Methods: Monitoring and data management

Data monitoring

An independent TSC will be responsible for oversight of the trial in order to safeguard the interests of trial participants. The TSC will provide advice to the chief investigator, NCTU, and the trial sponsor (University of East Anglia). A separate DMC comprising three independent researchers (one statistician) will monitor adverse event and adverse reactions. They will undertake a mid-recruitment review of adverse event and adverse reaction data. Their report will be reviewed by the TSC.

Adverse events

For peer review only - http://bmjopen.bmj.com/site/about/guidelines.xhtml

Adverse events refer to unwanted medical events (for example, worsening symptoms) occurring throughout the trial, regardless of whether they are causally related to the trial procedures. For the purposes of this trial the following would be considered adverse events: increase in extent of self-harm or suicidal ideation; significant worsening in symptoms (i.e. an increase in CRIES-8 score equal to or greater than seven); and/or concern regarding decline in mental state. Precautions have been taken to reduce the likelihood of such events occurring; for example the therapists delivering the CT-PTSD sessions will be trained in how to manage any distress that arises. Therapists in both arms of treatment will have experience in working with complex populations and management of risk. A Safety Management Plan detailing procedures for dealing with adverse events and adverse reactions has been agreed with NCTU.

Public and Patient Involvement (PPI)

The original trial design was formulated with input from several PPI groups. Service users commenting on the PYCES trial (NIHR RfPB-funded RCT addressing PTSD in preschool children) also stressed the need to investigate to youth with PTSD following multiple trauma exposure. Patient/participant feedback was an essential component of a case series of the treatment protocol (CT-PTSD) for PTSD in this group. Families participating in this study provided extensive feedback on the acceptability of the treatment protocol and the research procedures developed for use with this multiple trauma population. These responses were collated and were used to guide the development of this protocol.

The youth panel of *inspire* (a PPI group hosted by Norfolk and Suffolk NHS Foundation Trust [NSFT]) identified several agencies to involve in the service study and dissemination, and raised questions over the nature of "treatment as usual" for this population and the adequate "dosage" of CT-PTSD. The inspire youth panel were keen to be represented on the TMG and TSC. The panel were positive about the long-term benefits that may arise from this treatment, the CAMHS setting and the close involvement of PPI groups.

For peer review only - http://bmjopen.bmj.com/site/about/guidelines.xhtml

RMS met with the UK Clinical Research Network Young Person's Mental Health Advisory Group (YPMHAG) to receive their views on the initial proposed trial design. They raised issues about on-going post-trial care for trial participants, and the consenting procedure (addressed in the ethics section of this proposal). They were also concerned about psychotherapy being delivered poorly or in an overly-generic way. Group members commented that despite sometimes being disclosed to mental health teams, traumatic experiences were often not addressed in treatment or even mentioned again.

PPI representatives, recruited from the *inspire* group, contributed to the development of the study participant information sheets and consent forms. PPI representatives will be recruited to the Trial Management Group, will meet with the trial team on a regular basis to provide more specific input on study procedures, and will assist with writing the final report. Trial results will be communicated to all participants through a newsletter.

Ethics and dissemination

Research ethics approval and protocol amendments

DECRYPT was approved by a UK Health Research Authority (HRA) Research Ethics Committee (REC; East of England – Cambridge South, 16/EE/0233). The study personnel, a Trial Management Group, a TSC and a DMC, have been established and will ensure that the study is conducted within appropriate NHS and professional ethical guidelines. Good Clinical Practice training will have been undertaken by all those directly involved in running the study. Protocol amendments will require approval from the REC, and where relevant will be passed on to the trial register (i.e. ISRCTN).

Consent and assent

For youth aged under 16, informed consent will be provided by parents and caregivers, and the child or young person will also be asked to give their assent for trial entry. Youth aged 16 years

For peer review only - http://bmjopen.bmj.com/site/about/guidelines.xhtml

or older can provide informed consent without their parent or caregiver's involvement. Additional consent is required for the audio recording of therapy sessions. Additional consent forms will also be used for qualitative data collection, to ensure that participants agree to the interviews being recorded and their anonymised data (i.e. quotes) being used in future publications.

Confidentiality

The trial database will be password protected and only accessible to members of the DECRYPT trial team at NCTU, and external regulators if requested. The servers are protected by firewalls and are patched and maintained according to best practice. The physical location of the servers is protected by CCTV and security door access. Data will be entered in the approved DECRYPT database by a member of the DECRYPT trial team at NCTU or by assessors at each site, and protected using established NCTU procedures.

The identification, screening and enrolment logs, linking participant identifiable data to the pseudoanonymised Participant Identification Number, will be held locally by the trial team. This will either be held in written form in a locked filing cabinet or electronically in password protected form on secure computers. After completion of the trial the identification, screening and enrolment logs will be stored securely by the sites for 10 years unless otherwise advised by NCTU.

Declaration of interests

Some investigators in DECRYPT provide training in the delivery of CT-PTSD, for which they receive payment (e.g. to clinical psychology or cognitive behavioural therapy training courses, conference workshops).

Dissemination policy

There are no publication restrictions and findings will be disseminated broadly to participants, healthcare professionals, the public, and other relevant groups. The study findings will be published in peer-reviewed journals. Clinical workshops will be offered to practitioners and team

For peer review only - http://bmjopen.bmj.com/site/about/guidelines.xhtml

leads to share study findings and consider how practice can be improved. The full trial protocol is available from RMS.

Discussion

PTSD in children and adolescents represents a significant public health burden. It is hoped that the DECRYPT trial will provide insight into how some of the most severe PTSD cases in youth might be better treated in a real world setting, and provide a platform to future pragmatic research in this area.

For peer review only

For peer review only - http://bmjopen.bmj.com/site/about/guidelines.xhtml

Declarations

Ethics approval and consent to participate

DECRYPT was approved by a UK Health Research Authority (HRA) Research Ethics Committee (REC; 16/EE/0233). Informed consent is required for all participants in this trial.

Consent for publication

Not applicable.

Availability of data and material

The datasets generated during this trial will be available from the corresponding author on reasonable request. Data will be made available for those participants who have agreed to anonymous data sharing when giving their consent.

Competing interests

PS authored a treatment manual that is being used in this trial.

Funding

DECRYPT is funded by a National Institute for Health Research (NIHR) Career Development Fellowship to RMS (CDF-2015-08-073). This publication presents independent research funded by the National Institute for Health Research (NIHR). The views expressed are those of the author(s) and not necessarily those of the NHS, the NIHR or the Department of Health and Social Care. The NIHR had no role in the design of the study, or the collection, analysis, and interpretation of data, or in writing the manuscript.

Trial Sponsor: University of East Anglia. The sponsor had no role in the design of the study, or the collection, analysis, and interpretation of data, or in writing the manuscript.

Authors' contributions

RMS, LA, TD, PS, NM, PS, AD, JW, LS and SB each contributed to the design of the trial. RMS, LA, LG, KT, JC, PS, NM, PS, EB, AP, AH, JW, BG, GMD, MH, PAA, DT and ES oversaw recruitment and data collection. LA, RMS, ES and MH drafted the protocol. All authors read and

For peer review only - http://bmjopen.bmj.com/site/about/guidelines.xhtml

approved the final manuscript. All authors have agreed both to be personally accountable for the author's own contributions and to ensure that questions related to the accuracy or integrity of any part of the work, even ones in which the author was not personally involved, are appropriately investigated, resolved, and the resolution documented in the literature.

Acknowledgements

The authors gratefully acknowledge the support of the Trial Steering Committee (Sam Cartwright-Hatton, Peter Fonagy, Shirley Reynolds and William Yule) and Data Monitoring Committee members (Barney Dunn, Ian Goodyer and Victoria Vickerstaff). We are very grateful for the important contribution of all the young people and carers who helped to shape this study as it was being designed.

For peer review only

For peer review only - http://bmjopen.bmj.com/site/about/guidelines.xhtml

References

1. Cohen JA. Practice parameters for the assessment and treatment of children and adolescents with posttraumatic stress disorder. J Am Acad Child Adolesc Psychiatry 1998;**37**(10, Suppl):4S-26S.

2. Gillies D, Taylor F, Gray C, et al. Psychological therapies for the treatment of post-traumatic stress disorder in children and adolescents. The Cochrane database of systematic reviews 2012;**12**:CD006726.

3. Morgan L, Scourfield J, Williams D, et al. The Aberfan disaster: 33-year follow-up of survivors. Br J Psychiatry 2003;**182**(6):532-36.

4. Yule W, Bolton D, Udwin O, et al. The long-term psychological effects of a disaster experienced in adolescence: I: The incidence and course of PTSD. J Child Psychol Psychiatry 2000;**41**(4):503-11.

5. Psychiatrists RCo. *Building and sustaining specialist CAMHS to improve outcomes for children and young people*. London: Royal College of Psychiatrists, 2013.

6. Lewis SJ, Arseneault L, Caspi A, et al. The epidemiology of trauma and post-traumatic stress disorder in a representative cohort of young people in England and Wales. The lancet Psychiatry 2019;**6**(3):247-56.

7. American Psychiatric Association. *The Diagnostic and Statistical Manual of Mental Disorders, fifth edition*. Arlington: American Psychiatric Association, 2013.

8. Landolt MA, Schnyder U, Maier T, et al. Trauma exposure and posttraumatic stress disorder in adolescents: a national survey in Switzerland. J Trauma Stress 2013;**26**(2):209-16.

9. Copeland WE, Keeler G, Angold A, et al. Traumatic events and posttraumatic stress in childhood. Arch Gen Psychiat 2007;**64**(5):577-84.

For peer review only - http://bmjopen.bmj.com/site/about/guidelines.xhtml

10. Fletcher KE. Childhood posttraumatic stress disorder. In: Mash EJ, Barkley RA, eds. Child Psychopathology, 2nd Edition. New York, NY: The Guildford Press, 2003:330-71.

11. Suliman S, Mkabile SG, Fincham DS, et al. Cumulative effect of multiple trauma on symptoms of posttraumatic stress disorder, anxiety, and depression in adolescents. Compr Psychiatry 2009;**50**(2):121-7.

12. Holt MK, Finkelhor D, Kantor GK. Multiple victimization experiences of urban elementary school students: associations with psychosocial functioning and academic performance. Child abuse & neglect 2007;**31**(5):503-15.

13. Banyard VL, Williams LM, Siegel JA. The long-term mental health consequences of child sexual abuse: an exploratory study of the impact of multiple traumas in a sample of women. J Trauma Stress 2001;**14**(4):697-715.

14. Spertus IL, Yehuda R, Wong CM, et al. Childhood emotional abuse and neglect as predictors of psychological and physical symptoms in women presenting to a primary care practice. Child Abuse and Neglect 2003;**27**(11):1247-58.

15. Cloitre M, Stolbach BC, Herman JL, et al. A developmental approach to complex PTSD: childhood and adult cumulative trauma as predictors of symptom complexity. J Trauma Stress 2009;**22**(5):399-408.

16. Silverman AB, Reinherz HZ, Giaconia RM. The long-term sequelae of child and adolescent abuse: a longitudinal community study. Child abuse & neglect 1996;**20**(8):709-23.

17. Arnow BA. Relationships between childhood maltreatment, adult health and psychiatric outcomes, and medical utilization. J Clin Psychiatry 2004;**65 Suppl 12**:10-5.

18. Lansford JE, Dodge KA, Pettit GS, et al. A 12-year prospective study of the long-term effects of early child physical maltreatment on psychological, behavioral, and academic problems in adolescence. Archives of Pediatrics and Adolescent Medicine 2002;**156**(8):824-30.

For peer review only - http://bmjopen.bmj.com/site/about/guidelines.xhtml

19. Kerig PK, Ward RM, Vanderzee KL, et al. Posttraumatic stress as a mediator of the relationship between trauma and mental health problems among juvenile delinquents. J Youth Adolesc 2009;**38**(9):1214-25.

20. Ruchkin V, Henrich CC, Jones SM, et al. Violence exposure and psychopathology in urban youth: the mediating role of posttraumatic stress. J Abnorm Child Psychol 2007;**35**(4):578-93.

21. Campbell R, Greeson MR, Bybee D, et al. The co-occurrence of childhood sexual abuse, adult sexual assault, intimate partner violence, and sexual harassment: a mediational model of posttraumatic stress disorder and physical health outcomes. J Consult Clin Psychol 2008;**76**(2):194-207.

22. Lang AJ, Aarons GA, Gearity J, et al. Direct and indirect links between childhood maltreatment, posttraumatic stress disorder, and women's health. Behavioral medicine (Washington, DC) 2008;**33**(4):125-35.

23. Cloitre M, Cohen LR, Edelman RE, et al. Posttraumatic stress disorder and extent of trauma exposure as correlates of medical problems and perceived health among women with childhood abuse. Women and Health 2001;**34**(3):1-17.

24. Subica AM, Claypoole KH, Wylie AM. PTSD'S mediation of the relationships between trauma, depression, substance abuse, mental health, and physical health in individuals with severe mental illness: evaluating a comprehensive model. Schizophrenia research 2012;**136**(1-3):104-9.

25. National Institute for Clinical Excellence. *Post-traumatic stress disorder (PTSD): the treatment of PTSD in adults and children*. London: NICE, 2005.

For peer review only - http://bmjopen.bmj.com/site/about/guidelines.xhtml

26. Bisson JI, Ehlers A, Matthews R, et al. Psychological treatments for chronic post-traumatic stress disorder. Systematic review and meta-analysis. The British journal of psychiatry : the journal of mental science 2007;**190**:97-104.

27. Powers MB, Halpern JM, Ferenschak MP, et al. A meta-analytic review of prolonged exposure for posttraumatic stress disorder. Clin Psychol Rev 2010;**30**(6):635-41.

28. Hoppen TH, Morina N. Is high-quality of trials associated with lower treatment efficacy? A meta-analysis on the association between study quality and effect sizes of psychological interventions for pediatric PTSD. Clin Psychol Rev 2020;**78**:101855.

29. Meiser-Stedman R, McKinnon A, Dixon C, et al. A core role for cognitive processes in the acute onset and maintenance of post-traumatic stress in children and adolescents. J Child Psychol Psychiatry 2019;**60**(8):875-84.

30. Smith P, Perrin S, Yule W, et al. *Post traumatic stress disorder: Cognitive therapy with children and young people*. London: Routledge, 2009.

31. Meiser-Stedman R, Smith P, McKinnon A, et al. Cognitive therapy as an early treatment for post-traumatic stress disorder in children and adolescents: a randomized controlled trial addressing preliminary efficacy and mechanisms of action. J Child Psychol Psychiatry 2017;**58**(5):623-33.

32. Smith P, Yule W, Perrin S, et al. Cognitive-behavioral therapy for PTSD in children and adolescents: a preliminary randomized controlled trial. J Am Acad Child Adolesc Psychiatry 2007;**46**(8):1051-61.

33. Sachser C, Keller F, Goldbeck L. Complex PTSD as proposed for ICD-11: validation of a new disorder in children and adolescents and their response to Trauma-Focused Cognitive Behavioral Therapy. J Child Psychol Psychiatry 2017;**58**(2):160-68.

For peer review only - http://bmjopen.bmj.com/site/about/guidelines.xhtml

34. Chan AW, Tetzlaff JM, Altman DG, et al. SPIRIT 2013 statement: defining standard protocol items for clinical trials. Ann Intern Med 2013;**158**(3):200-7.

35. Perrin S, Meiser-Stedman R, Smith P. The Children's Revised Impact of Event Scale (CRIES): Validity as a screening instrument for PTSD. Behav Cogn Psychoth 2005;**33**(4):487-98.

36. Foa EB, McLean CP, Capaldi S, et al. Prolonged exposure vs supportive counseling for sexual abuse–related PTSD in adolescent girls: a randomized clinical trial. JAMA 2013;**310**(24):2650-57.

37. Sachser C, Berliner, L., Holt, T., Jensen, T., Jungbluth, N., Risch, E., Rosner, R., Goldbeck, L. International development and validation of the Child and Adolescent Trauma Screening Questionnaire (CATS). International Society for Traumatic Stress Studies. Dallas, USA, 2016.

38. Chorpita BF, Moffitt CE, Gray J. Psychometric properties of the Revised Child Anxiety and Depression Scale in a clinical sample. Behav Res Ther 2005;**43**(3):309-22.

39. Costello EJ, Angold A. Scales to Assess Child and Adolescent Depression - Checklists, Screens, and Nets. J Am Acad Child Adolesc Psychiatry 1988;**27**(6):726-37.

40. Stringaris A, Goodman R, Ferdinando S, et al. The Affective Reactivity Index: a concise irritability scale for clinical and research settings. J Child Psychol Psychiatry 2012;**53**(11):1109-17.

41. Shaffer D, Gould MS, Brasic J, et al. A children's global assessment scale (CGAS). Arch Gen Psychiatry 1983;**40**(11):1228-31.

42. Goodman R. Psychometric properties of the strengths and difficulties questionnaire. J Am Acad Child Adolesc Psychiatry 2001;**40**(11):1337-45.

43. Goodman M, Patil U, Triebwasser J, et al. Parental burden associated with borderline personality disorder in female offspring. J Pers Disord 2011;**25**(1):59-74.

For peer review only

For peer review only - http://bmjopen.bmj.com/site/about/guidelines.xhtml

44. Kroenke K, Spitzer RL, Williams JB. The PHQ-9: validity of a brief depression severity measure. J Gen Intern Med 2001;**16**(9):606-13.

45. Spitzer RL, Kroenke K, Williams JBW, et al. A brief measure for assessing generalized anxiety disorder: the GAD-7. Archives of Internal Medicine 2006;**166**(10):1092-97.

46. Foa EB, McLean CP, Zang Y, et al. Psychometric Properties of the Posttraumatic Diagnostic Scale for DSM-5 (PDS-5). Psychol Assess 2015.

47. Byford S, Barrett B, Roberts C, et al. Cost-effectiveness of selective serotonin reuptake inhibitors and routine specialist care with and without cognitive–behavioural therapy in adolescents with major depression. The British Journal of Psychiatry 2007;**191**(6):521-27.

48. Wille N, Badia X, Bonsel G, et al. Development of the EQ-5D-Y: a child-friendly version of the EQ-5D. Quality of life research : an international journal of quality of life aspects of treatment, care and rehabilitation 2010;**19**(6):875-86.

49. Anilmis JV, Stewart CS, Roddy S, et al. Understanding the relationship between schematic beliefs, bullying, and unusual experiences in 8-14 year olds. European psychiatry : the journal of the Association of European Psychiatrists 2015;**30**(8):920-3.

50. Zimet GD, Dahlem NW, Zimet SG, et al. The Multidimensional Scale of Perceived Social Support. J Pers Assess 1988;**52**(1):30 - 41.

51. Meiser-Stedman R, Smith P, Bryant R, et al. Development and validation of the Child Post-Traumatic Cognitions Inventory (CPTCI). J Child Psychol Psychiatry 2009;**50**(4):432-40.

52. Meiser-Stedman R, Smith P, Yule W, et al. The Trauma Memory Quality Questionnaire: preliminary development and validation of a measure of trauma memory characteristics for children and adolescents. Memory 2007;**15**(3):271-9.

For peer review only - http://bmjopen.bmj.com/site/about/guidelines.xhtml

53. Meiser-Stedman R, Shepperd A, Glucksman E, et al. Thought control strategies and rumination in youth with acute stress disorder and posttraumatic stress disorder following single-event trauma. J Child Adolesc Psychopharmacol 2014;**24**(1):47-51.

54. Alberici A, Meiser-Stedman R, Claxton J, et al. The Preliminary Development and Validation of a Trauma-Related Safety-Seeking Behavior Measure for Youth: The Child Safety Behavior Scale (CSBS). J Trauma Stress 2018;**31**(5):643-53.

55. Ehlers A, Clark DM, Hackmann A, et al. A randomized controlled trial of cognitive therapy, a self-help booklet, and repeated assessments as early interventions for posttraumatic stress disorder. Arch Gen Psychiat 2003;**60**(10):1024-32.

56. Shirk SR, Saiz CC. Clinical, empirical, and developmental perspectives on the therapeutic relationship in child psychotherapy. Dev Psychopathol 1992;**4**(04):713-28.

57. Guest G, Bunce A, Johnson L. How many interviews are enough? An experiment with data saturation and variability. Field methods 2006;**18**(1):59-82.

58. Cary CE, McMillen JC. The data behind the dissemination: a systematic review of trauma-focused cognitive behavioral therapy for use with children and youth. Children and Youth Services Review 2012;**34**(4):748-57.

59. Gutermann J, Schreiber F, Matulis S, et al. Psychological Treatments for Symptoms of Posttraumatic Stress Disorder in Children, Adolescents, and Young Adults: A Meta-Analysis. Clin Child Fam Psychol Rev 2016;**19**(2):77-93.

60. Braun V, Clarke V. Using thematic analysis in psychology. Qualitative research in psychology 2006;**3**(2):77-101.

61. Barry CA, Britten N, Barber N, et al. Using reflexivity to optimize teamwork in qualitative research. Qualitative health research 1999;**9**(1):26-44.

For peer review only - http://bmjopen.bmj.com/site/about/guidelines.xhtml

62. National Institute for Health and Care Excellence. *Guide to the methods of technology appraisal*. London: NICE, 2013.

63. Drummond MF, Sculpher MJ, Claxton K, et al. *Methods for the economic evaluation of health care programmes*: Oxford university press, 2015.

64. Thompson SG, Barber JA. How should cost data in pragmatic randomised trials be analysed? Bmj 2000;**320**(7243):1197-200.

65. Manca A, Hawkins N, Sculpher MJ. Estimating mean QALYs in trial-based cost-effectiveness analysis: the importance of controlling for baseline utility. Health economics 2005;**14**(5):487-96.

66. Briggs AH. A Bayesian approach to stochastic cost-effectiveness analysis. Health economics 1999;**8**(3):257-61.

67. Fenwick E, Byford S. A guide to cost-effectiveness acceptability curves. The British Journal of Psychiatry 2005;**187**(2):106-08.

68. Assmann SF, Pocock SJ, Enos LE, et al. Subgroup analysis and other (mis) uses of baseline data in clinical trials. The Lancet 2000;**355**(9209):1064-69.

For peer review only

For peer review only - http://bmjopen.bmj.com/site/about/guidelines.xhtml

		STUDY PERIOD					
	Screening	Baseline	Allocation	Post-allocation			
TIMEPOINT	$-t_1$	$-t_2$	0	*Mid [2.5m]*	*Post [5m]*	*Follow up [11m]*	*Follow up [29m]*
ENROLMENT:							
Eligibility screen	X						
Informed consent		X					
PTSD severity (CRIES-8)		X		X	X	X	X
PTSD diagnosis (CPSS-I-5)		X			X	X	X
Allocation			X				
INTERVENTIONS:							
CT-PTSD			←	↔	→		
TAU			←	↔	→		
ASSESSMENTS:							
Demographics & life history		X					
PTSD severity, DSM-5 (CATS)		X		X	X	X	X
Anxiety & depression (RCADS)		X			X	X	X
Suicidal ideation (MFQ)		X			X		
Affect regulation (ARI)		X			X	X	
Functioning (CGAS)		X			X	X	X
Parent/caregiver-report SDQ		X			X	X	X
Borderline traits (MSI-BPD-C)		X			X	X	
Caregiver depression (PHQ-9)		X			X	X	
Caregiver anxiety (GAD-7)		X			X	X	
Caregiver post-traumatic stress (PDS-5)		X			X	X	
Health & social care resource use (CA-SUS)		X			X	X	
Health-related quality of life (EQ-5D-Y)		X			X	X	
Voices (interview)		X			X		
Trauma-related appraisals (CPTCI)		X		X	X		
Trauma memory quality (TMQQ)		X		X	X		
Rumination & self-blame		X		X	X		
Safety-seeking behaviours (CSBS)		X		X	X		
Social support (MSPSS)		X		X	X		
Treatment credibility scale				X	X		
Therapeutic alliance (TASC-r)				X	X		
Adverse events		X			X	X	

Figure 1. Standard Protocol Items: Recommendations for Interventional Trials (SPIRIT) diagram detailing trial activities and measures and their timing.

259x334mm (300 x 300 DPI)

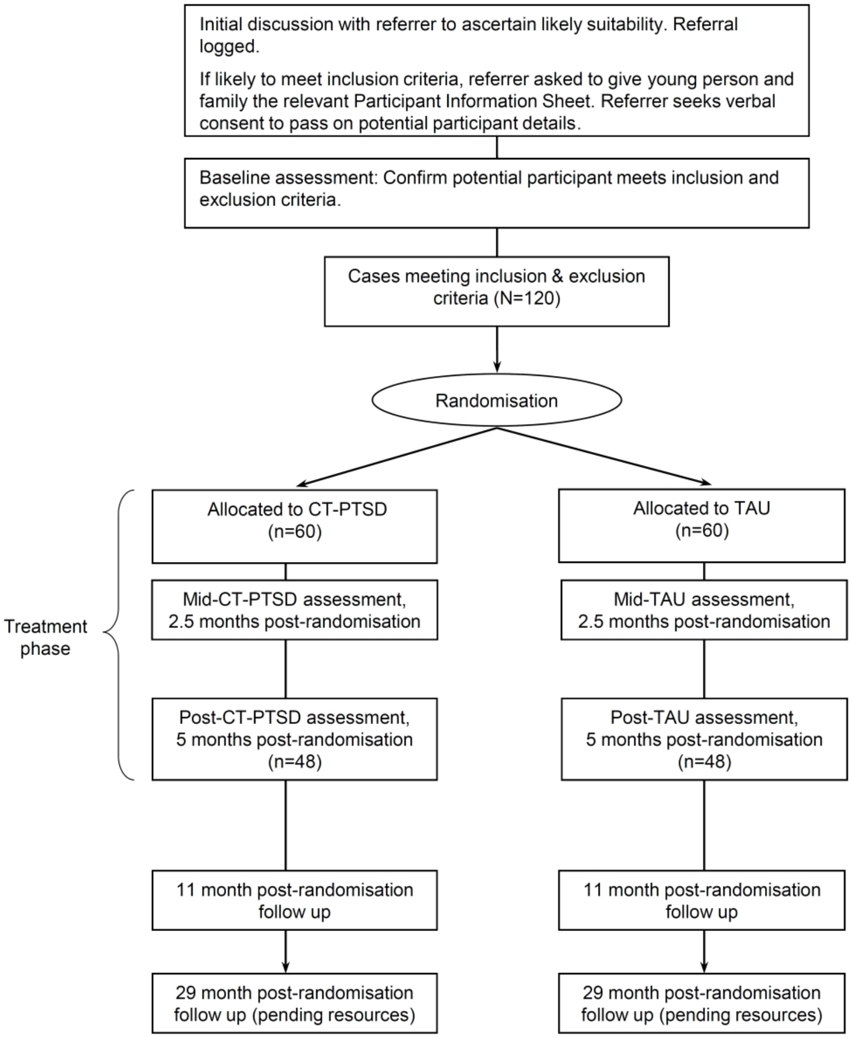

Figure 2. Trial Consolidated Standards of Reporting Trials (CONSORT) diagram

380x400mm (300 x 300 DPI)

STANDARD PROTOCOL ITEMS: RECOMMENDATIONS FOR INTERVENTIONAL TRIALS

SPIRIT 2013 Checklist: Recommended items to address in a clinical trial protocol and related documents*

Section/item	ItemNo	Description	Page
**Administrative information**			
Title	1	Descriptive title identifying the study design, population, interventions, and, if applicable, trial acronym	1
Trial registration	2a	Trial identifier and registry name. If not yet registered, name of intended registry	3
	2b	All items from the World Health Organization Trial Registration Data Set	4
Protocol version	3	Date and version identifier	4
Funding	4	Sources and types of financial, material, and other support	23
Roles and responsibilities	5a	Names, affiliations, and roles of protocol contributors	23
	5b	Name and contact information for the trial sponsor	23
	5c	Role of study sponsor and funders, if any, in study design; collection, management, analysis, and interpretation of data; writing of the report; and the decision to submit the report for publication, including whether they will have ultimate authority over any of these activities	23
	5d	Composition, roles, and responsibilities of the coordinating centre, steering committee, endpoint adjudication committee, data management team, and other individuals or groups overseeing the trial, if applicable (see Item 21a for data monitoring committee)	17, 19, 20, 24
**Introduction**			
Background and rationale	6a	Description of research question and justification for undertaking the trial, including summary of relevant studies (published and unpublished) examining benefits and harms for each intervention	5
	6b	Explanation for choice of comparators	5-8

For peer review only

Objectives	7	Specific objectives or hypotheses	8
Trial design	8	Description of trial design including type of trial (eg, parallel group, crossover, factorial, single group), allocation ratio, and framework (eg, superiority, equivalence, noninferiority, exploratory)	8-9
**Methods: Participants, interventions, and outcomes**			
Study setting	9	Description of study settings (eg, community clinic, academic hospital) and list of countries where data will be collected. Reference to where list of study sites can be obtained	9
Eligibility criteria	10	Inclusion and exclusion criteria for participants. If applicable, eligibility criteria for study centres and individuals who will perform the interventions (eg, surgeons, psychotherapists)	9
Interventions	11a	Interventions for each group with sufficient detail to allow replication, including how and when they will be administered	10-11
	11b	Criteria for discontinuing or modifying allocated interventions for a given trial participant (eg, drug dose change in response to harms, participant request, or improving/worsening disease)	n/a
	11c	Strategies to improve adherence to intervention protocols, and any procedures for monitoring adherence (eg, drug tablet return, laboratory tests)	11
	11d	Relevant concomitant care and interventions that are permitted or prohibited during the trial	11
Outcomes	12	Primary, secondary, and other outcomes, including the specific measurement variable (eg, systolic blood pressure), analysis metric (eg, change from baseline, final value, time to event), method of aggregation (eg, median, proportion), and time point for each outcome. Explanation of the clinical relevance of chosen efficacy and harm outcomes is strongly recommended	11-14
Participant timeline	13	Time schedule of enrolment, interventions (including any run-ins and washouts), assessments, and visits for participants. A schematic diagram is highly recommended (see Figure)	14; Fig 1 (p35)

For peer review only - http://bmjopen.bmj.com/site/about/guidelines.xhtml

Sample size	14	Estimated number of participants needed to achieve study objectives and how it was determined, including clinical and statistical assumptions supporting any sample size calculations	14
Recruitment	15	Strategies for achieving adequate participant enrolment to reach target sample size	15
**Methods: Assignment of interventions (for controlled trials)**			
Allocation:			
Sequence generation	16a	Method of generating the allocation sequence (eg, computer-generated random numbers), and list of any factors for stratification. To reduce predictability of a random sequence, details of any planned restriction (eg, blocking) should be provided in a separate document that is unavailable to those who enrol participants or assign interventions	16
Allocation concealment mechanism	16b	Mechanism of implementing the allocation sequence (eg, central telephone; sequentially numbered, opaque, sealed envelopes), describing any steps to conceal the sequence until interventions are assigned	16
Implementation	16c	Who will generate the allocation sequence, who will enrol participants, and who will assign participants to interventions	16
Blinding (masking)	17a	Who will be blinded after assignment to interventions (eg, trial participants, care providers, outcome assessors, data analysts), and how	16
	17b	If blinded, circumstances under which unblinding is permissible, and procedure for revealing a participant's allocated intervention during the trial	n/a
**Methods: Data collection, management, and analysis**			
Data collection methods	18a	Plans for assessment and collection of outcome, baseline, and other trial data, including any related processes to promote data quality (eg, duplicate measurements, training of assessors) and a description of study instruments (eg, questionnaires, laboratory tests) along with their reliability and validity, if known. Reference to where data collection forms can be found, if not in the protocol	16*17
	18b	Plans to promote participant retention and complete follow-up, including list of any outcome data to be collected for participants who discontinue or deviate from intervention protocols	n/a

For peer review only - http://bmjopen.bmj.com/site/about/guidelines.xhtml

Data management	19	Plans for data entry, coding, security, and storage, including any related processes to promote data quality (eg, double data entry; range checks for data values). Reference to where details of data management procedures can be found, if not in the protocol	17
Statistical methods	20a	Statistical methods for analysing primary and secondary outcomes. Reference to where other details of the statistical analysis plan can be found, if not in the protocol	17
	20b	Methods for any additional analyses (eg, subgroup and adjusted analyses)	18-19
	20c	Definition of analysis population relating to protocol non-adherence (eg, as randomised analysis), and any statistical methods to handle missing data (eg, multiple imputation)	n/a
**Methods: Monitoring**			
Data monitoring	21a	Composition of data monitoring committee (DMC); summary of its role and reporting structure; statement of whether it is independent from the sponsor and competing interests; and reference to where further details about its charter can be found, if not in the protocol. Alternatively, an explanation of why a DMC is not needed	19-20
	21b	Description of any interim analyses and stopping guidelines, including who will have access to these interim results and make the final decision to terminate the trial	19-20
Harms	22	Plans for collecting, assessing, reporting, and managing solicited and spontaneously reported adverse events and other unintended effects of trial interventions or trial conduct	20
Auditing	23	Frequency and procedures for auditing trial conduct, if any, and whether the process will be independent from investigators and the sponsor	n/a
**Ethics and dissemination**			
Research ethics approval	24	Plans for seeking research ethics committee/institutional review board (REC/IRB) approval	20

Protocol amendments	25	Plans for communicating important protocol modifications (eg, changes to eligibility criteria, outcomes, analyses) to relevant parties (eg, investigators, REC/IRBs, trial participants, trial registries, journals, regulators)	20
Consent or assent	26a	Who will obtain informed consent or assent from potential trial participants or authorised surrogates, and how (see Item 32)	21
	26b	Additional consent provisions for collection and use of participant data and biological specimens in ancillary studies, if applicable	21
Confidentiality	27	How personal information about potential and enrolled participants will be collected, shared, and maintained in order to protect confidentiality before, during, and after the trial	21
Declaration of interests	28	Financial and other competing interests for principal investigators for the overall trial and each study site	21
Access to data	29	Statement of who will have access to the final trial dataset, and disclosure of contractual agreements that limit such access for investigators	n/a
Ancillary and post-trial care	30	Provisions, if any, for ancillary and post-trial care, and for compensation to those who suffer harm from trial participation	n/a
Dissemination policy	31a	Plans for investigators and sponsor to communicate trial results to participants, healthcare professionals, the public, and other relevant groups (eg, via publication, reporting in results databases, or other data sharing arrangements), including any publication restrictions	21
	31b	Authorship eligibility guidelines and any intended use of professional writers	n/a
	31c	Plans, if any, for granting public access to the full protocol, participant-level dataset, and statistical code	n/a
**Appendices**			
Informed consent materials	32	Model consent form and other related documentation given to participants and authorised surrogates	n/a
Biological specimens	33	Plans for collection, laboratory evaluation, and storage of biological specimens for genetic or molecular analysis in the current trial and for future use in ancillary studies, if applicable	n/a

For peer review only

*It is strongly recommended that this checklist be read in conjunction with the SPIRIT 2013 Explanation & Elaboration for important clarification on the items. Amendments to the protocol should be tracked and dated. The SPIRIT checklist is copyrighted by the SPIRIT Group under the Creative Commons "Attribution-NonCommercial-NoDerivs 3.0 Unported" license.

For peer review only

BMJ Open

The DECRYPT trial: Study protocol for a phase II randomised controlled trial of cognitive therapy for post-traumatic stress disorder (PTSD) in youth exposed to multiple traumatic stressors.

Journal:	*BMJ Open*
Manuscript ID	bmjopen-2020-047600.R1
Article Type:	Protocol
Date Submitted by the Author:	30-Apr-2021

Complete List of Authors:	Allen, Leila; University of East Anglia, Department of Clinical Psychology and Psychological Therapies, Norwich Medical School<br>Ashford, Polly-Anna; University of East Anglia, Norwich Clinical Trials Unit, Norwich Medical School<br>Beeson, Ella; Hertfordshire Partnership University NHS Foundation Trust<br>Byford, Sarah; King's College London, Centre for the Economics of Mental and Physical Health<br>Chow, Jessica; University of East Anglia, Department of Clinical Psychology and Psychological Therapies, Norwich Medical School<br>Dalgleish, Tim; MRC Cognition and Brain Sciences Unit; Cambridgeshire and Peterborough NHS Foundation Trust<br>Danese, Andrea; King's College London Institute of Psychiatry Psychology and Neuroscience, Department of Child and Adolescent Psychiatry; King's College London, Social, Genetic and Developmental Psychiatry Centre<br>Finn, Jack; University of East Anglia, Department of Clinical Psychology and Psychological Therapies, Norwich Medical School<br>Goodall, Ben; North East London NHS Foundation Trust<br>Grainger, Lauren; University of East Anglia, Department of Clinical Psychology and Psychological Therapies, Norwich Medical School<br>Hammond, Matthew ; University of East Anglia, Norwich Clinical Trials Unit, Norwich Medical School<br>Humphrey, Ayla; Cambridgeshire and Peterborough NHS Foundation Trust<br>Mahoney-Davies, Gerwyn; Cardiff and Vale University Health Board<br>Morant, Nicola; University College London,<br>Shepstone, Lee; University of East Anglia, Norwich Clinical Trials Unit, Norwich Medical School<br>Sims, Erika; University of East Anglia, Norwich Clinical Trials Unit, Norwich Medical School<br>Smith, Patrick; King's College London Institute of Psychiatry Psychology and Neuroscience, Department of Psychology<br>Stallard, Paul; University of Bath, Department for Health<br>Swanepoel, Annie; Hertfordshire Partnership University NHS Foundation Trust<br>Trickey, David ; Anna Freud Centre<br>Trigg, Katie; University of East Anglia, Department of Clinical Psychology and Psychological Therapies, Norwich Medical School

	Wilson, Jon; Norfolk and Suffolk NHS Foundation Trust,<br>Meiser-Stedman , Richard ; University of East Anglia, Department of Clinical Psychology and Psychological Therapies
**Primary Subject Heading**:	Mental health
Secondary Subject Heading:	Mental health
Keywords:	Child & adolescent psychiatry < PSYCHIATRY, Anxiety disorders < PSYCHIATRY, Depression & mood disorders < PSYCHIATRY

SCHOLARONE™
Manuscripts

I, the Submitting Author has the right to grant and does grant on behalf of all authors of the Work (as defined in the below author licence), an exclusive licence and/or a non-exclusive licence for contributions from authors who are: i) UK Crown employees; ii) where BMJ has agreed a CC-BY licence shall apply, and/or iii) in accordance with the terms applicable for US Federal Government officers or employees acting as part of their official duties; on a worldwide, perpetual, irrevocable, royalty-free basis to BMJ Publishing Group Ltd ("BMJ") its licensees and where the relevant Journal is co-owned by BMJ to the co-owners of the Journal, to publish the Work in this journal and any other BMJ products and to exploit all rights, as set out in our licence.

The Submitting Author accepts and understands that any supply made under these terms is made by BMJ to the Submitting Author unless you are acting as an employee on behalf of your employer or a postgraduate student of an affiliated institution which is paying any applicable article publishing charge ("APC") for Open Access articles. Where the Submitting Author wishes to make the Work available on an Open Access basis (and intends to pay the relevant APC), the terms of reuse of such Open Access shall be governed by a Creative Commons licence – details of these licences and which Creative Commons licence will apply to this Work are set out in our licence referred to above.

Other than as permitted in any relevant BMJ Author's Self Archiving Policies, I confirm this Work has not been accepted for publication elsewhere, is not being considered for publication elsewhere and does not duplicate material already published. I confirm all authors consent to publication of this Work and authorise the granting of this licence.

For peer review only

The DECRYPT trial: Study protocol for a phase II randomised controlled trial of cognitive therapy for post-traumatic stress disorder (PTSD) in youth exposed to multiple traumatic stressors.

Leila Allen[1], Polly-Anna Ashford[2], Ella Beeson[3], Sarah Byford[4], Jessica Chow[1], Tim Dalgleish[5,6], Andrea Danese[7,8], Jack Finn[1], Ben Goodall[9], Lauren Grainger[1], Matt Hammond[2], Ayla Humphrey[6], Gerwyn Mahoney-Davies[10], Nicola Morant[11], Lee Shepstone[2], Erika Sims[2], Patrick Smith[12], Paul Stallard[13], Annie Swanepoel[3]; David Trickey[14]; Katie Trigg[1], Jon Wilson[15], Richard Meiser-Stedman[1]

[1] Department of Clinical Psychology & Psychological Therapies, Norwich Medical School, University of East Anglia

[2] Norwich Clinical Trials Unit, Norwich Medical School, University of East Anglia

[3] Hertfordshire Partnership Foundation NHS Trust

[4] King's Health Economics, King's College London, London

[5] Medical Research Council Cognition and Brain Sciences Unit, University of Cambridge

[6] Cambridgeshire and Peterborough NHS Foundation Trust

[7] Social, Genetic and Developmental Psychiatry Centre, King's College London

[8] Department of Child and Adolescent Psychiatry, Institute of Psychiatry, Psychology and Neuroscience, King's College London

[9] North East London NHS Foundation Trust

[10] Cardiff & Vale University Health Board

[11] Division of Psychiatry, Faculty of Brain Sciences, University College London

For peer review only - http://bmjopen.bmj.com/site/about/guidelines.xhtml

[12] Department of Psychology, Institute of Psychiatry, Psychology and Neuroscience, King's College London

[13] Department for Health, University of Bath

[14] Anna Freud National Centre for Children and Families, London

[15] Norfolk & Suffolk Foundation NHS Trust

Corresponding author:

Richard Meiser-Stedman, Department of Clinical Psychology & Psychological Therapies, Norwich Medical School, University of East Anglia, email: r.meiser-stedman@uea.ac.uk

For peer review only - http://bmjopen.bmj.com/site/about/guidelines.xhtml

Abstract

Background: Post-traumatic stress disorder (PTSD) is a distressing and disabling condition that affects significant numbers of children and adolescents. Youth exposed to multiple traumas (e.g. abuse, domestic violence) are at particular risk of developing PTSD. Cognitive-Therapy for PTSD (CT-PTSD), derived from adult work, is a theoretically-informed, disorder-specific form of trauma-focused cognitive-behavioural therapy (TF-CBT). While efficacious for child and adolescent single-event trauma samples, its effectiveness in routine settings with more complex, multiple-trauma exposed youth has not been established. The Delivery of Cognitive Therapy for Young People after Trauma (DECRYPT) randomised controlled trial (RCT) examines the effectiveness of CT-PTSD for treating PTSD following multiple trauma exposure in children and young people in comparison to treatment as usual (TAU).

Methods/Design: This protocol describes a two-arm, patient-level, single blind, superiority RCT comparing CT-PTSD (n=60) to TAU (n=60) in children and young people aged 8-17 years with a diagnosis of PTSD following multiple trauma exposure. The primary outcome is PTSD severity assessed using the Children's Revised Impact of Event Scale (8-item version) at post-treatment (i.e. approximately five months post-randomisation). Secondary outcomes include structured interview assessment for PTSD, complex PTSD symptoms, depression and anxiety, overall functioning and parent-rated mental health. Mid-treatment and 11- and 29-month post-randomisation assessments will also be completed. Process-outcome evaluation will consider which mechanisms underpin or moderate recovery. Qualitative interviews with the young people, their families and their therapists will be undertaken. Cost-effectiveness of CT-PTSD relative to TAU will be also be assessed.

Ethics and dissemination: This trial protocol has been approved by a UK Health Research Authority (HRA) Research Ethics Committee (East of England – Cambridge South, 16/EE/0233). Findings will be disseminated broadly via peer-reviewed empirical journal articles, conference presentations, and clinical workshops.

For peer review only - http://bmjopen.bmj.com/site/about/guidelines.xhtml

Trial registration: ISRCTN, ISRCTN12077707. Registered 24th October 2016, http://www.isrctn.com/ISRCTN12077707. Trial recruitment commenced on the 1st February 2017. It is anticipated that recruitment will continue until June 2021, with 11 month assessments being concluded in May 2022.

For peer review only

For peer review only - http://bmjopen.bmj.com/site/about/guidelines.xhtml

Strengths and limitations of this study

- This study will involve a highly pragmatic evaluation of a psychological therapy (Cognitive Therapy for post-traumatic stress disorder [PTSD]) for children and young people with PTSD, being embedded in front-line UK National Health Service child and adolescent mental health services (CAMHS).
- The trial will include youth with the more severe, multiple trauma histories typically seen in CAMHS, and in addition to questionnaire and interview measures of PTSD, the trial will consider Complex PTSD symptoms.
- The trial will explore putative mediators and moderators of treatment outcome, and also includes a cost-effectiveness evaluation and qualitative data collection.
- There is no standard "treatment as usual" for this population, and it is anticipated that treatment in this control arm will vary.

For peer review only

For peer review only - http://bmjopen.bmj.com/site/about/guidelines.xhtml

Administrative information

Scientific title: Cognitive Therapy for the treatment of post-traumatic stress disorder (PTSD) in youth exposed to multiple traumatic stressors: a phase II randomised controlled trial

Trial registration: ISRCTN12077707

Keywords: Post traumatic stress disorder, Children, Young people, PTSD, Cognitive therapy, multiple trauma.

Trial status: Open to recruitment (Opened 1st March 2017; anticipated recruitment end date, 31st May 2021)

Protocol version: 4.0 (15th April 2020)

Word count: 4876

For peer review only

For peer review only - http://bmjopen.bmj.com/site/about/guidelines.xhtml

Introduction

Background

Posttraumatic stress disorder (PTSD) is a deeply distressing and disabling psychiatric disorder. PTSD in youth is usually comorbid with other psychiatric conditions,[1 2] and may persist for years or even decades if untreated [3 4]. The UK Royal College of Psychiatrists estimates[5] the prevalence of PTSD in UK youth to be 3%, while a recent epidemiological study suggested that over 7% of UK youth will have developed PTSD at some point by the age of 18.[6]

Traumatic events, defined[7] as those that involve exposure to actual or threatened death, serious injury, or sexual violence, are experienced by up to two-thirds of children by age 16.[8 9] A significant minority of trauma-exposed children and adolescents go on to develop posttraumatic stress disorder (PTSD).[1 10] For those young people subjected to *multiple* traumatic stressors (e.g. physical or sexual abuse, witnessing domestic violence, war or community violence), the clinical presentation of PTSD is particularly severe.[11 12] The life-long impact of multiple trauma exposure in childhood is well documented, with dramatically increased prevalence of a range of emotional disorders, compromised educational, occupational and social functioning, increased use of mental health services and poor physical health.[13-18] PTSD has been shown to have a uniquely harmful role in determining the long-term response to childhood trauma exposure, by mediating the relationship between such experiences and a range of physical and mental health outcomes later in childhood[19 20] and adulthood[21-23], including psychosis.[24]

In adults, empirical support for trauma-focused cognitive behaviour therapy (TF-CBT) is substantial.[25-27] TF-CBT typically comprises several core elements: psychoeducation, exposure (to help desensitise patients to trauma memories), cognitive elements (to reframe the meanings and interpretations associated with trauma and its aftermath), and coping management (e.g. problem-solving, anxiety management). Over the past 15 years several randomised controlled trials have been conducted that support the efficacy of TF-CBT (using a variety of specific manuals) in children and

For peer review only - http://bmjopen.bmj.com/site/about/guidelines.xhtml

adolescents.[28] While this evidence shows considerable promise, a number of important issues remain to be resolved.

First, existing trials rarely compare an experimental treatment to treatment as usual, i.e. active treatment/clinical care from a child and adolescent mental health service. Moreover, many studies have not focused on cases of the kind typically referred to mental health services, instead focusing on youth identified in child protection/social services settings. Similarly, many trials have not used "front-line therapists", reducing the ability to implement trial findings in routine settings.

Second, the cost-effectiveness of implementing a trauma-focused psychological intervention (such as different forms of TF-CBT) in youth mental health services has received little consideration. Evaluating the health economic implications of the investment of time in training and supervising therapists in delivering such specific treatments is essential for the future service delivery.

Third, it is important to consider whether a cognitive therapy for PTSD (CT-PTSD) treatment protocol, a particular form of TF-CBT, can be utilised in child and adolescent mental health services by front-line therapists. Step increases in treatment efficacy for adults with PTSD have been achieved through careful individual formulation and the enhanced use of theoretically-derived techniques in CT-PTSD.[29-31] The cognitive model of PTSD has been largely supported in children and youth.[32] While CT-PTSD adapted for youth[33 34] has been shown to be efficacious for PTSD following single event trauma when delivered in research clinics[34 35], its effectiveness in routine clinical settings with youth exposed to multiple traumas has yet to be established. We felt that CT-PTSD may translate well to "front-line" clinical settings as it employs a formulation-based approach, i.e. clinicians are able to tailor session content (i.e. particular techniques) to their client's individual presentation which may be particularly helpful when working with clients with more complex histories.

Fourth, the responsiveness of Complex PTSD symptoms in children and adolescents to psychological treatment is still poorly understood. Symptoms of Complex PTSD include the defining

For peer review only - http://bmjopen.bmj.com/site/about/guidelines.xhtml

criteria of PTSD alongside disruptions in emotion regulation, relational capacities and self-concept, and are associated with multiple trauma exposures (e.g. sexual or physical abuse). While the understanding of Complex PTSD in adults has seen significant advances over recent years (with the disorder now included in ICD-11), little is known about the nature and presentation of Complex PTSD in youth, and our treatments for these youth lag behind provision for adults. The ability of youth with high levels of Complex PTSD symptoms to derive benefit from psychological therapies for PTSD is largely unknown. However, the first study to address this issue in youth found that Complex PTSD symptoms were responsive to TF-CBT, and that the presence of Complex PTSD did not lessen the efficacy of TF-CBT to treat PTSD symptoms.[36]

Fifth, moderators and mediators of treatment responsiveness are still poorly understood. While change in negative trauma-related misappraisals has been shown to underpin (i.e. mediate) treatment response for cognitive-behavioural therapies[34 35] (hence our specific secondary hypothesis regarding this mechanism), other potential mechanisms or moderating factors have received scant or no attention. Potential contraindicative moderators for cognitive-behavioural therapies for PTSD in youth have received comparatively little attention. Shedding light on these factors would inform decision making about treatments for youth with PTSD and clarify the essential "ingredients" of successful treatment and barriers to change.

Sixth, the lived experiences of youth undergoing treatment for PTSD following multiple traumas has received little consideration. Qualitative methods may offer important insights into the acceptability and feasibility of delivering treatments like CT-PTSD for youth with PTSD. Such insights may direct how treatments are evaluated and delivered in the future, as well as informing clinician attitudes to the management of this population's needs.

Objectives

This study aims to fill these gaps in the literature by addressing several objectives. The primary objective of the study is to evaluate whether CT-PTSD is an effective treatment for PTSD

For peer review only - http://bmjopen.bmj.com/site/about/guidelines.xhtml

symptoms in 8-17 year old youth who have been exposed to multiple traumatic stressors, relative to TAU in UK National Health Service (NHS) child and adolescent/youth mental health services. The primary outcome is the Child Revised Impact of Event Scale (8-item version; CRIES-8) score at post-treatment. The CRIES-8 is the routine outcome monitoring measure for PTSD in UK child and adolescent mental health services (CAMHS). We hypothesised that CT-PTSD will be superior to TAU at post-treatment (approximately five months post-randomisation) with respect to scores on the CRIES-8. The secondary objectives of the study are as follows: (1) is CT-PTSD effective in the treatment of complex PTSD, anxiety, depression, general functioning and parent-related mental health in youth with PTSD, relative to TAU? (2) Is the treatment cost-effective, relative to TAU? (3) If effective relative to TAU, by what mechanisms does CT-PTSD have its effect? (4) What demographic, symptomatic, cognitive and psychosocial factors moderate response to treatment? (5) What are the views and experiences of youth with PTSD, their parents, and therapists, about receiving or delivering CT-PTSD, and how do these inform judgements of the acceptability and feasibility of CT-PTSD? In particular, we hypothesised that CT-PTSD would be superior to TAU with respect to complex PTSD symptoms, anxiety, depression, general functioning and parent-related mental health in youth with PTSD; CT-PTSD would be cost-effective relative to TAU; and that the efficacy of CT-PTSD would be mediated through change in trauma-related appraisals.

Given the significant uncertainties over the effect size associated with both CT-PTSD and TAU in this context and the ability of therapists in settings to deliver CT-PTSD with this relatively complex population, this study was considered to be a Phase II trial. It is anticipated that the trial will inform the development of a later definitive trial of CT-PTSD for this population.

Study design

This study is a two-arm, patient-level, single blind, superiority RCT comparing CT-PTSD to treatment-as-usual (TAU). Participants will be allocated to CT-PTSD or TAU according to a 1:1 ratio, with stratification by baseline CRIES-8score and recruiting site.

For peer review only - http://bmjopen.bmj.com/site/about/guidelines.xhtml

This protocol has been written in accordance with the Standard Protocol Items: Recommendations for Interventional Trials (SPIRIT) 2013 statement.[37]

Methods: Participants, interventions, and outcomes

Study setting

Trial data collection, randomisation, blinding and data analysis will be overseen by the Norwich Clinical Trials Unit (NCTU). Participants will be recruited from NHS CAMHS and youth mental health services in England and Wales.

Eligibility criteria

A total of 120 children/young people aged 8-17 years will be randomised to either CT-PTSD (n=60) or TAU (n=60). Youth are eligible to be included in the study if they: (1) meet the criteria for a diagnosis of PTSD, as defined by the Diagnostic and Statistical Manual of Mental Disorders, Fifth Edition (DSM-5);[7] (2) score 17 or greater on the CRIES-8; and (3) have been exposed to multiple traumatic stressors.

Exclusion criteria are: (1) *change* of prescribed psychiatric medication within the past two months (though receiving medication was not an exclusion criterion); (2) pervasive developmental disorder or neurodevelopmental disorder (e.g. autism, but not ADHD); (3) intellectual disability; (4) another primary psychiatric diagnosis or clinical need that warrants treatment ahead of PTSD (e.g. psychosis, suicidal behaviour, conduct disorder); (5) inability to speak English; (6) on-going exposure to threat (e.g. living with an abuser; regularly placing self in danger) or safeguarding issues; (7) strong likelihood of being unable to complete treatment (e.g. imminent house or foster placement move); or (8) history of organic brain damage.

Interventions

For peer review only - http://bmjopen.bmj.com/site/about/guidelines.xhtml

CT-PTSD. CT-PTSD for PTSD is a structured, fully manualised [33] psychological treatment delivered in an individual format for children and adolescents. The proposed frequency and duration of treatment is up to 15 treatment sessions (typically 10-12, of 60-90 minutes duration).

CT-PTSD involves several core elements: psychoeducation, with an emphasis on the role of cognitive processes in the onset and maintenance of PTSD; narrative work and imaginal reliving to help develop a coherent trauma narrative; cognitive restructuring (to reframe the meanings and interpretations associated with trauma and its aftermath), and coping management (e.g. addressing maladaptive strategies such as thought suppression, rumination and safety-seeking behaviours). Up to three sessions will be allowed for stabilising other comorbid conditions and difficulties (e.g. depression or self-harm).

CT-PTSD will be delivered by NHS CAMHS/youth mental health service therapists who will have completed training in CT-PTSD by a member of the trial team. Trial therapists who deliver CT-PTSD must have an appropriate professional qualification (e.g. as a nurse, occupational therapist, clinical psychologist, social worker, psychiatrist or BABCP-registered cognitive-behavioural therapist) and be approved by their local site Principal Investigator to act as a trial therapist. Individual teams will nominate staff members to complete the CT-PTSD training.

CT-PTSD will be delivered wherever is permitted and feasible for local therapists (e.g. in NHS mental health clinics, local GP surgeries, at home). Supervision will be provided by a clinical psychologist from the trial team. Following the completion of a course of CT-PTSD, usual NHS care arrangements will apply for participants in this arm.

TAU. Mental health professionals and others involved in the care of the participants in the TAU arm will be encouraged to provide whatever help they deem necessary, e.g. general clinical management, supportive counselling, family therapy, medication. TAU must involve an active treatment, i.e. it cannot involve being on a waiting list. Since TF-CBT has been a recommended treatment for PTSD in youth in the UK since 2005,[25] no therapists in the TAU arm were prevented

For peer review only - http://bmjopen.bmj.com/site/about/guidelines.xhtml

from delivering this intervention; they were not, however, trained by the research team to deliver the CT-PTSD intervention. Therapist contact in the TAU arm would not be prescribed by trial participation in any way, with one exception: the participants will receive no contact with the trained trial therapists delivering CT-PTSD, and their therapist/clinician will not receive supervision for that case from a trained trial therapist.

Treatment integrity

Trial therapists will be supervised by members of the trial team while working with trial participants. Trial therapists delivering CT-PTSD will record sessions with participants and rate the use of specific treatment techniques in notes and recording sheets. Trial collaborators will oversee the quality assurance of the training and will monitor and ensure treatment adherence.

Outcomes

The timing of all measures and assessments is presented in Figure 1.

Primary clinical outcome. The primary outcome measure is PTSD severity using the Child Revised Impact of Events Scale (CRIES-8) score at post-treatment (approximately five months post-randomisation). The CRIES-8, a validated self-report questionnaire,[38] is the routine outcome monitoring tool for PTSD in children and adolescents endorsed by the UK Children and Young People's programme – Improving Access to Psychological Therapies (CYP-IAPT). Selecting this measure as our primary outcome is therefore consistent with the pragmatic, "front-line" focus of the present trial, and will allow clinicians to interpret the trial's findings with regards to their routine outcome measures. The CRIES-8 has the advantage of being used internationally in numerous languages, and being impervious to future changes in diagnostic algorithms, having been in constant use for over 25 years. The CRIES-8 will also be completed at baseline, 2.5 months post-randomisation (mid-treatment), and 11 months and 29 months post-randomisation.

Secondary clinical outcomes. PTSD diagnosis and symptoms using the Child PTSD Symptom Scale for DSM-5, interviewer version (CPSS-I-5; structured interview, psychometric

For peer review only - http://bmjopen.bmj.com/site/about/guidelines.xhtml

properties made available by authors),[39] with additional items for measuring dissociation and complex PTSD (that is, items addressing disruptions in emotion regulation, relational capacities and self-concept, that are unique to complex PTSD). The Child and Adolescent Trauma Screen (CATS) will also be used as a self-report questionnaire to address all DSM-5 PTSD symptoms,[40] with additional items measuring dissociation and complex PTSD. Anxiety and depression will be assessed using the Revised Child Anxiety and Depression Scale (RCADS).[41] Suicidal ideation will be assessed using five items from the Mood and Feelings Questionnaire (MFQ).[42] Affect regulation and irritability will be assessed by the Affective Reactivity Index (ARI; child and parent/caregiver-report versions).[43] Clinician-rated general functioning will be assessed using the Children's Global Assessment Scale (CGAS).[44] Parent/caregiver-rated mental health and well-being will be indexed by the Strengths and Difficulties Questionnaire (SDQ).[45] Parent/caregiver-rated borderline personality traits will be assessed using the McLean Screening Instrument for Borderline Personality Disorder, caregiver version (MSI-BPD-C).[46]

In order to ascertain whether parent/caregiver mental health moderates their children's ability to benefit from intervention, and whether parent/caregiver mental health improve with their child receiving intervention, clinical outcomes for parents/caregivers will be assessed. Parent/caregiver depression, anxiety and post-traumatic stress will be assessed using the Patient Health Questionnaire (PHQ-9),[47] Generalised Anxiety Disorder Assessment (GAD-7),[48] and Posttraumatic Stress Diagnostic Scale, DSM-5 version (PDS-5),[49] respectively.

Health economic evaluation measures. Resource use will be collected using the Child and Adolescent Service Use Schedule (CA-SUS),[50] modified for a PTSD population, a structured economic interview for youth mental health populations. The CA-SUS will be administered by trial team members at baseline and trained assessors (blinded to allocation) at post-treatment and 11 month follow up assessments. The interview involves both the young person (if aged 12 or above) and the young person's primary parent/caregiver. Effectiveness will be measured using the youth

For peer review only - http://bmjopen.bmj.com/site/about/guidelines.xhtml

version of the EuroQol measure of health related quality of life (EQ-5D-Y)[51], a preference-based, generic measure capable of generating quality adjusted life years (QALYs).

Process measures. Items from an existing interview[52] will be used to assess whether a young person is experiencing hearing voices. Social support will be assessed using the Multidimensional Scale of Perceived Social Support.[53] Several mechanisms proposed by cognitive theories for PTSD will be measured: trauma-related appraisals (the Children's Post-Traumatic Cognitions Inventory, CPTCI),[54] trauma memory quality (the Trauma Memory Quality Scale, TMQQ),[55] trauma-related rumination and self-blame[56] and safety-seeking behaviours (the Child Safety Behaviour Scale, CSBS).[57] Treatment credibility will be assessed using four items derived from a previous trial.[30] Therapeutic alliance will be assessed using the Therapeutic Alliance Scale for Children, revised (TASC-r; completed by participant).[58]

Qualitative data. Semi-structured interviews will be conducted to explore the perspectives of youth receiving CT-PTSD, family members, and therapists who deliver CT-PTSD. Interviews topic guides will be tailored to each of these perspectives, adapted to be age- appropriate for youth respondents, and reviewed by Patient and Public Involvement (PPI) representatives. They will explore experiences and perceived impacts of treatment and its constituent components; the acceptability and feasibility of the treatment received / delivered; and views of being involved in a randomised controlled trial and the assessment processes involved. Interviews with youth will be conducted after their post-treatment assessment. In line with sample size guidelines for qualitative interviews,[59] sample sizes of 12-15 for youth and family member interviews are planned, sampled to include youth across the trial age spectrum. A similar sample size is planned for trial therapists.

Participant timeline

Participants will be assessed five times during the study: baseline, mid-treatment (approximately 2.5 months post-randomisation), post-treatment (approximately five months post-

For peer review only - http://bmjopen.bmj.com/site/about/guidelines.xhtml

randomisation) and at 11- and 29-month post-randomisation follow up assessments (approximately 6- and 24-months post-treatment; see Figure 1).

Sample size

This is the first trial to compare CT-PTSD (or any other treatment) to routine care in NHS CAMHS and youth mental health settings for children and young people with PTSD following multiple trauma exposure. While two waiting-list controlled trials have been conducted of the CT-PTSD treatment package in research clinics,[34 35] no UK trials have evaluated the effectiveness of routine care for the treatment of PTSD in young people with multiple trauma exposure, or the effectiveness of CT-PTSD as delivered by therapists working in routine clinical settings. As such, no reliable estimates of effect size are available. An attempt was made to estimate likely effect size based on meta-analyses of existing psychological therapies for PTSD in children and adolescents. The waiting-list controlled effect size of CT-PTSD was greater than 1.2 in each of the studies to have examined this intervention to date.[34 35] The Cochrane meta-analysis of treatments for PTSD in children and adolescents[10] yielded an effect size of 1.05 for all psychological therapies versus control conditions, whereas for CBT versus control conditions, the effect size was 1.34. However, these effect sizes are likely to overestimate the efficacy of CBT; few studies were included in the headline analyses, and those that were included were waiting-list controlled, rather than using an active control condition (e.g. TAU or other interventions that involved clinician/therapist contact). A high quality trial of another psychological therapy (prolonged exposure, a form of TF-CBT) for adolescent girls with PTSD following sexual abuse, that used supportive counselling as a control intervention, yielded a controlled (i.e. between groups) effect size of 1.0.[39] An alternative meta-analysis,[60] including a broader range of studies to evaluate CBT in youth with PTSD (many of which used active control treatments such as supportive counselling) suggested a controlled effect size of around 0.67; a more recent meta-analysis[61] suggested an effect size for TF-CBT of .79. The more conservative effect size estimate of .67 was used for the proposed trial.

For peer review only - http://bmjopen.bmj.com/site/about/guidelines.xhtml

In order to have 90% power to detect a between groups effect size of 0.67 (two-tailed t-test, 0.05 significance level) a sample size of 96 (48 participants per group) is required; a combined sample size of 96 would have 80% power to detect an effect size of .58 (two-tailed t-test, 0.05 significance level). In order to account for drop out (estimated at 20%), 120 participants will be recruited.

Recruitment

Participants will be recruited from NHS Trusts in England and Wales. Clinicians in participating teams will be encouraged to contact the trial team to discuss potential participants to ensure that they meet inclusion criteria. If a child or young person appears eligible, the clinician will share information with the participant and their family about the study who will be given at least 48 hours to decide whether they wish to participate. Consenting families will be contacted by the trial team to discuss further and to arrange baseline assessments; trial eligibility will be confirmed following this assessment. Children and young people will not be able to enter DECRYPT unless a therapist within the team is available to offer CT-PTSD and another clinician/therapist is available to offer TAU, i.e. TAU cannot involve a waiting list.

Methods: Assignment of interventions

Allocation

Following pre-trial assessments, consenting participants will be randomised to study arms stratified by CRIES-8 score (17-28, 29-40) and site (i.e. NHS Trust). This cut-off for stratification was based on data from an unpublished pilot study that preceded DECRYPT, where the mean score for study participants was 28.75. An online randomisation service managed by NCTU will assign allocation to groups. Allocation is by pre-set lists of permuted blocks with randomly distributed block sizes (determined by the trial statistician). The lists will be generated by the Data Management Team at NCTU. The trial manager will enrol participants. Following randomisation, participants,

For peer review only - http://bmjopen.bmj.com/site/about/guidelines.xhtml

For peer review only

their clinical team and their General Practitioner (GP) will be notified of their allocation by the trial manager. See Figure 2 below for the Consolidated Standards of Reporting Trials (CONSORT) diagram.

Blinding

Trained assessors collecting post-treatment and follow-up data will be blinded to group allocation. These assessments will be undertaken by trained assessors with no other role in the trial. Following allocation to CT-PTSD or TAU, all participants in the study, their care coordinator/ referrer and clinical team (if applicable) are asked not to reveal the group to which the participants were randomised to the trained assessor.

Methods: Data collection, management, and analysis

Data collection methods

Quantitative outcome variable data will be collected using paper forms and entered electronically on to the trial database, or online. Qualitative data will be collected using face-to-face interviews, or where this is not possible telephone or video-call interviews. An appropriately trained individual will undertake semi-structured interviews with participants and their families after treatment has been completed. For participants that have withdrawn from treatment or the trial but consent to taking part in a qualitative interview, this will be completed when appropriate. Interviews will be audio-recorded and transcribed verbatim, with all identifying information removed.

Data management

To maintain confidentiality, each participant will be given a unique trial Participant Identification Number (PIN). Data will be entered under this identification number onto the central database stored on the servers based at NCTU. The database and coding frames have been developed by the Clinical Trial Manager in conjunction with NCTU. The database software provides a number of features to help maintain data quality, including maintaining an audit trail, allowing custom

For peer review only - http://bmjopen.bmj.com/site/about/guidelines.xhtml

validations on all data, allowing users to raise data query requests, and search facilities to identify validation failure/ missing data. After completion of the trial, the database will be retained on the servers of NCTU for on-going analysis of secondary outcomes.

Statistical methods

A full statistical analysis plan (SAP) will be written and agreed with the Data Monitoring Committee (DMC) and Trial Steering Committee (TSC) prior to database lock and any data analysis. This plan will be amended only with agreement of the former two committees.

Our primary analysis will compare CT-PTSD with TAU on CRIES-8 scores at post-treatment. The primary analysis will be on the intention to treat principal: i.e. all participants will be followed up for data collection irrespective of adherence to treatment and will be analysed according to group allocation rather than intervention received. Assuming a normal distribution (potentially of transformed values), a linear model will be constructed. This will include recruiting site (as a random factor), CRIES-8 at baseline (as a covariate) and any factors considered prognostic and determined in advance of any analysis, together with treatment arm as a fixed effect. Analysis of other secondary outcomes, and of putative moderators and mediators, will be considered exploratory.

The primary intention to treat analysis is intended to provide inferences regarding the effectiveness of the intervention overall not to provide inferences regarding the causal effect of the intervention itself, but on the intervention as deployed in 'real life'. Statistical significance will be set at the conventional (two-tailed) 5% level and all parameter estimates will be presented with 95% confidence intervals. Analyses will be carried out by the trial statistician blinded to group identity, (i.e. 'subgroup' blind). There are no plans for interim efficacy or subgroup analyses. Analyses will be carried out in SAS (currently version 9.4).

Mediation and moderation analyses will be undertaken following the procedures outlined by Kraemer and colleagues.[62] As with all our secondary analyses, these will be considered exploratory.

Qualitative analysis

For peer review only - http://bmjopen.bmj.com/site/about/guidelines.xhtml

Qualitative data will be analysed using thematic analysis within NVivo software.[63] A collaborative approach throughout the analytic process in which emerging themes are discussed between those who collect data and other members of the research team will enhance reflexivity and validity.[64] The views and experiences of each group (children/young people, parents/caregivers, trial therapists) will be analysed both separately and in comparison to each other in order to gain a multi-perspective view of the acceptability and feasibility of delivering CT-PTSD in routine NHS child and youth mental health services. Both commonalities and variations within and between these stakeholder groups will be explored.

Health economics evaluation

Economic evaluation will compare CT-PTSD with TAU at the 11-month follow-up. It will take the NHS/personal social services perspective, preferred by NICE,[65] but will additionally include education services, given the age of the population. Service use data will be collected using the CA-SUS while health-related quality of life will be assessed using the EQ-5D-Y (see above). Data on CT-PTSD contacts and TAU (CAMHS) contacts and on indirect time for the intervention (e.g. supervision, training) will be collected directly from therapists/clinicians and service records; participants will be asked not to mention any CAMHS/mental health contacts so as to reduce the possibility of unblinding and double-counting of service contacts. Service use estimates will be combined with standard UK sources for unit costs to estimate total costs (including NHS reference costs for hospital contacts, the British National Formulary for medications and the PSSRU Unit Costs of Health and Social Care). The cost of CT-PTSD will be directly calculated.[66]

Economic analysis will be carried out on an intention-to-treat basis using an analysis plan to be drawn up prior to data analysis. Costs and outcomes will be compared and presented in terms of mean differences and 95% confidence intervals obtained by non-parametric bootstrap regression to account for the non-normal distribution commonly found in economic data.[67] Cost-effectiveness will be assessed through the calculation of incremental cost-effectiveness ratios (the additional cost of

For peer review only - http://bmjopen.bmj.com/site/about/guidelines.xhtml

one intervention compared with another divided by the additional effects). The primary analysis will focus on effects measured using the CRIES-8, known to be sensitive to change in this population. Secondary analyses will explore cost-effectiveness in terms of QALYs derived from the EQ-5D-Y and using the area under the curve approach,[68] a more policy relevant measure that is preferred by NICE, but with unknown sensitivity in young people with PTSD. Uncertainty will be explored using cost-effectiveness planes and cost-effectiveness acceptability curves based on the net-benefit approach.[69 70] These curves are an alternative to confidence intervals around ICERs and show the probability that one intervention is cost-effective compared to the other, for a range of values that a decision maker would be willing to pay for an additional unit of an outcome. All economic analyses will be adjusted for baseline CRIES-8 score and site, in line with the clinical analyses, plus baseline values of the variables of interest (cost, QALYs), to provide a more relevant treatment-effect estimate.[71]

Methods: Monitoring and data management

Data monitoring

An independent TSC will be responsible for oversight of the trial in order to safeguard the interests of trial participants. The TSC will provide advice to the chief investigator, NCTU, and the trial sponsor (University of East Anglia). A separate DMC comprising three independent researchers (one statistician) will monitor adverse event and adverse reactions. They will undertake a mid-recruitment review of adverse event and adverse reaction data. Their report will be reviewed by the TSC.

Adverse events

Adverse events refer to unwanted medical events (for example, worsening symptoms) occurring throughout the trial, regardless of whether they are causally related to the trial procedures. For the purposes of this trial the following would be considered adverse events: increase in extent of

For peer review only - http://bmjopen.bmj.com/site/about/guidelines.xhtml

self-harm or suicidal ideation; significant worsening in symptoms (i.e. an increase in CRIES-8 score equal to or greater than seven); and/or concern regarding decline in mental state. Precautions have been taken to reduce the likelihood of such events occurring; for example the therapists delivering the CT-PTSD sessions will be trained in how to manage any distress that arises. Therapists in both arms of treatment will have experience in working with complex populations and management of risk. A Safety Management Plan detailing procedures for dealing with adverse events and adverse reactions has been agreed with NCTU.

Public and Patient Involvement (PPI)

The original trial design was formulated with input from several PPI groups. Service users commenting on the PYCES trial (NIHR RfPB-funded RCT addressing PTSD in preschool children) also stressed the need to investigate to youth with PTSD following multiple trauma exposure. Patient/participant feedback was an essential component of a case series of the treatment protocol (CT-PTSD) for PTSD in this group. Families participating in this study provided extensive feedback on the acceptability of the treatment protocol and the research procedures developed for use with this multiple trauma population. These responses were collated and were used to guide the development of this protocol.

The youth panel of *inspire* (a PPI group hosted by Norfolk and Suffolk NHS Foundation Trust [NSFT]) identified several agencies to involve in the service study and dissemination, and raised questions over the nature of “treatment as usual” for this population and the adequate “dosage” of CT-PTSD. The inspire youth panel were keen to be represented on the TMG and TSC. The panel were positive about the long-term benefits that may arise from this treatment, the CAMHS setting and the close involvement of PPI groups.

RMS met with the UK Clinical Research Network Young Person’s Mental Health Advisory Group (YPMHAG) to receive their views on the initial proposed trial design. They raised issues about on-going post-trial care for trial participants, and the consenting procedure (addressed in the

For peer review only - http://bmjopen.bmj.com/site/about/guidelines.xhtml

ethics section of this proposal). They were also concerned about psychotherapy being delivered poorly or in an overly-generic way. Group members commented that despite sometimes being disclosed to mental health teams, traumatic experiences were often not addressed in treatment or even mentioned again.

PPI representatives, recruited from the *inspire* group, contributed to the development of the study participant information sheets and consent forms. PPI representatives will be recruited to the Trial Management Group, will meet with the trial team on a regular basis to provide more specific input on study procedures, and will assist with writing the final report. Trial results will be communicated to all participants through a newsletter.

Ethics and dissemination

Research ethics approval and protocol amendments

DECRYPT was approved by a UK Health Research Authority (HRA) Research Ethics Committee (REC; East of England – Cambridge South, 16/EE/0233). The study personnel, a Trial Management Group, a TSC and a DMC, have been established and will ensure that the study is conducted within appropriate NHS and professional ethical guidelines. Good Clinical Practice training will have been undertaken by all those directly involved in running the study. Protocol amendments will require approval from the REC, and where relevant will be passed on to the trial register (i.e. ISRCTN). To date, protocol amendments have been made to: clarify CT-PTSD therapist background requirements, include screening of electronic notes in one NHS Trust, share core baseline assessment data with clinical teams in both arms of the trial (to avoid unnecessary duplication of assessments), provide clarification on where identifiable participant data would be stored, and clarify what would constitute an expected event rather than an adverse event or adverse reaction (major amendment, March 2017); removal of some secondary process outcome measures that felt to be overly burdensome for participants (intelligence testing, emotional regulation

For peer review only - http://bmjopen.bmj.com/site/about/guidelines.xhtml

strategies); clarifying that participants would receive payment for completing qualitative interviews in addition to the main trial assessments (major amendment, January 2018); and other procedural changes, such as trial team changes and the additional of students to the trial team. In light of the COVID-19 situation, an amendment was made to allow electronic (i.e. remote) consenting.

Consent and assent

For youth aged under 16, informed consent will be provided by parents and caregivers, and the child or young person will also be asked to give their assent for trial entry. Youth aged 16 years or older can provide informed consent without their parent or caregiver's involvement. Additional consent is required for the audio recording of therapy sessions. Additional consent forms will also be used for qualitative data collection, to ensure that participants agree to the interviews being recorded and their anonymised data (i.e. quotes) being used in future publications.

Confidentiality

The trial database will be password protected and only accessible to members of the DECRYPT trial team at NCTU, and external regulators if requested. The servers are protected by firewalls and are patched and maintained according to best practice. The physical location of the servers is protected by CCTV and security door access. Data will be entered in the approved DECRYPT database by a member of the DECRYPT trial team at NCTU or by assessors at each site, and protected using established NCTU procedures.

The identification, screening and enrolment logs, linking participant identifiable data to the pseudoanonymised Participant Identification Number, will be held locally by the trial team. This will either be held in written form in a locked filing cabinet or electronically in password protected form on secure computers. After completion of the trial the identification, screening and enrolment logs will be stored securely by the sites for 10 years unless otherwise advised by NCTU.

Declaration of interests

For peer review only - http://bmjopen.bmj.com/site/about/guidelines.xhtml

Some investigators in DECRYPT provide training in the delivery of CT-PTSD, for which they receive payment (e.g. to clinical psychology or cognitive behavioural therapy training courses, conference workshops).

Dissemination policy

There are no publication restrictions and findings will be disseminated broadly to participants, healthcare professionals, the public, and other relevant groups. The study findings will be published in peer-reviewed journals. Clinical workshops will be offered to practitioners and team leads to share study findings and consider how practice can be improved. The full trial protocol is available from RMS. Outcomes from the post-treatment and 11 month post-randomisation assessments may be analysed and published prior to the completion of the 29 month assessments.

Discussion

PTSD in children and adolescents represents a significant public health burden. It is hoped that the DECRYPT trial will provide insight into how some of the most severe PTSD cases in youth might be better treated in a real world setting, and provide a platform to future pragmatic research in this area.

For peer review only - http://bmjopen.bmj.com/site/about/guidelines.xhtml

Declarations

Ethics approval and consent to participate

DECRYPT was approved by a UK Health Research Authority (HRA) Research Ethics Committee (REC; 16/EE/0233). Informed consent is required for all participants in this trial.

Consent for publication

Not applicable.

Availability of data and material

The datasets generated during this trial will be available from the corresponding author on reasonable request. Data will be made available for those participants who have agreed to anonymous data sharing when giving their consent.

Competing interests

PS authored a treatment manual that is being used in this trial.

Funding

DECRYPT is funded by a National Institute for Health Research (NIHR) Career Development Fellowship to RMS (CDF-2015-08-073). This publication presents independent research funded by the National Institute for Health Research (NIHR). The views expressed are those of the author(s) and not necessarily those of the NHS, the NIHR or the Department of Health and Social Care. The NIHR had no role in the design of the study, or the collection, analysis, and interpretation of data, or in writing the manuscript.

Trial Sponsor: University of East Anglia. The sponsor had no role in the design of the study, or the collection, analysis, and interpretation of data, or in writing the manuscript.

Authors' contributions

RMS, LA, TD, PSm, NM, PSt, AD, JW, LS and SB each contributed to the design of the trial. RMS, LA, LG, KT, JC, PS, NM, PS, EB, AP, AH, JW, BG, GMD, MH, PA, DT, JF, AS and ES oversaw recruitment and data collection. LA, RMS, ES and MH drafted the protocol. All authors

For peer review only - http://bmjopen.bmj.com/site/about/guidelines.xhtml

read and approved the final manuscript. All authors have agreed both to be personally accountable for the author's own contributions and to ensure that questions related to the accuracy or integrity of any part of the work, even ones in which the author was not personally involved, are appropriately investigated, resolved, and the resolution documented in the literature.

Acknowledgements

The authors gratefully acknowledge the support of the Trial Steering Committee (Sam Cartwright-Hatton, Peter Fonagy, Shirley Reynolds and William Yule) and Data Monitoring Committee members (Barney Dunn, Ian Goodyer and Victoria Vickerstaff). We are very grateful for the important contribution of all the young people and carers who helped to shape this study as it was being designed.

For peer review only - http://bmjopen.bmj.com/site/about/guidelines.xhtml

References

1. Cohen JA. Practice parameters for the assessment and treatment of children and adolescents with posttraumatic stress disorder. *J Am Acad Child Adolesc Psychiatry* 1998;37(10, Suppl):4S-26S. doi: 10.1007/978-1-4613-4223-6_11 [published Online First: 1977/01/01]

2. Gillies D, Taylor F, Gray C, et al. Psychological therapies for the treatment of post-traumatic stress disorder in children and adolescents. *The Cochrane database of systematic reviews* 2012;12:CD006726. doi: 10.1002/14651858.CD006726.pub2 [published Online First: 2012/12/14]

3. Morgan L, Scourfield J, Williams D, et al. The Aberfan disaster: 33-year follow-up of survivors. *Br J Psychiatry* 2003;182(6):532-36. [published Online First: 1977/05/01]

4. Yule W, Bolton D, Udwin O, et al. The long-term psychological effects of a disaster experienced in adolescence: I: The incidence and course of PTSD. *J Child Psychol Psychiatry* 2000;41(4):503-11. doi: 10.1016/0006-2952(75)90412-8 [published Online First: 1975/10/15]

5. Psychiatrists RCo. Building and sustaining specialist CAMHS to improve outcomes for children and young people. London: Royal College of Psychiatrists 2013.

6. Lewis SJ, Arseneault L, Caspi A, et al. The epidemiology of trauma and post-traumatic stress disorder in a representative cohort of young people in England and Wales. *The lancet Psychiatry* 2019;6(3):247-56. doi: 10.1016/S2215-0366(19)30031-8 [published Online First: 2019/02/26]

7. American Psychiatric Association. The Diagnostic and Statistical Manual of Mental Disorders, fifth edition. Arlington: American Psychiatric Association 2013.

For peer review only - http://bmjopen.bmj.com/site/about/guidelines.xhtml

For peer review only

8. Landolt MA, Schnyder U, Maier T, et al. Trauma exposure and posttraumatic stress disorder in adolescents: a national survey in Switzerland. *J Trauma Stress* 2013;26(2):209-16. doi: 10.1002/jts.21794 [published Online First: 2013/03/16]

9. Copeland WE, Keeler G, Angold A, et al. Traumatic events and posttraumatic stress in childhood. *Arch Gen Psychiat* 2007;64(5):577-84. doi: 10.1001/archpsyc.64.5.577 [published Online First: 2007/05/09]

10. Fletcher KE. Childhood posttraumatic stress disorder. In: Mash EJ, Barkley RA, eds. Child Psychopathology, 2nd Edition. New York, NY: The Guildford Press 2003:330-71.

11. Suliman S, Mkabile SG, Fincham DS, et al. Cumulative effect of multiple trauma on symptoms of posttraumatic stress disorder, anxiety, and depression in adolescents. *Compr Psychiatry* 2009;50(2):121-7. doi: 10.1016/j.comppsych.2008.06.006 [published Online First: 2009/02/17]

12. Holt MK, Finkelhor D, Kantor GK. Multiple victimization experiences of urban elementary school students: associations with psychosocial functioning and academic performance. *Child abuse & neglect* 2007;31(5):503-15. doi: 10.1016/j.chiabu.2006.12.006 [published Online First: 2007/06/01]

13. Banyard VL, Williams LM, Siegel JA. The long-term mental health consequences of child sexual abuse: an exploratory study of the impact of multiple traumas in a sample of women. *J Trauma Stress* 2001;14(4):697-715. doi: 10.1023/a:1013085904337 [published Online First: 2002/01/05]

14. Spertus IL, Yehuda R, Wong CM, et al. Childhood emotional abuse and neglect as predictors of psychological and physical symptoms in women presenting to a primary care practice. *Child Abuse and Neglect* 2003;27(11):1247-58. doi: 10.1016/s0300-9629(75)80018-1 [published Online First: 1975/12/01]

For peer review only - http://bmjopen.bmj.com/site/about/guidelines.xhtml

15. Cloitre M, Stolbach BC, Herman JL, et al. A developmental approach to complex PTSD: childhood and adult cumulative trauma as predictors of symptom complexity. *J Trauma Stress* 2009;22(5):399-408. doi: 10.1002/jts.20444 [published Online First: 2009/10/02]

16. Silverman AB, Reinherz HZ, Giaconia RM. The long-term sequelae of child and adolescent abuse: a longitudinal community study. *Child abuse & neglect* 1996;20(8):709-23. doi: 10.1016/0145-2134(96)00059-2 [published Online First: 1996/08/01]

17. Arnow BA. Relationships between childhood maltreatment, adult health and psychiatric outcomes, and medical utilization. *J Clin Psychiatry* 2004;65 Suppl 12:10-5. [published Online First: 2004/08/19]

18. Lansford JE, Dodge KA, Pettit GS, et al. A 12-year prospective study of the long-term effects of early child physical maltreatment on psychological, behavioral, and academic problems in adolescence. *Archives of Pediatrics and Adolescent Medicine* 2002;156(8):824-30. [published Online First: 1976/02/01]

19. Kerig PK, Ward RM, Vanderzee KL, et al. Posttraumatic stress as a mediator of the relationship between trauma and mental health problems among juvenile delinquents. *J Youth Adolesc* 2009;38(9):1214-25. doi: 10.1007/s10964-008-9332-5 [published Online First: 2009/08/12]

20. Ruchkin V, Henrich CC, Jones SM, et al. Violence exposure and psychopathology in urban youth: the mediating role of posttraumatic stress. *J Abnorm Child Psychol* 2007;35(4):578-93. doi: 10.1007/s10802-007-9114-7 [published Online First: 2007/03/03]

21. Campbell R, Greeson MR, Bybee D, et al. The co-occurrence of childhood sexual abuse, adult sexual assault, intimate partner violence, and sexual harassment: a mediational model of posttraumatic stress disorder and physical health outcomes. *J Consult Clin Psychol* 2008;76(2):194-207. doi: 10.1037/0022-006x.76.2.194 [published Online First: 2008/04/02]

For peer review only

For peer review only - http://bmjopen.bmj.com/site/about/guidelines.xhtml

22. Lang AJ, Aarons GA, Gearity J, et al. Direct and indirect links between childhood maltreatment, posttraumatic stress disorder, and women's health. *Behavioral medicine (Washington, DC)* 2008;33(4):125-35. doi: 10.3200/bmed.33.4.125-136 [published Online First: 2008/03/05]

23. Cloitre M, Cohen LR, Edelman RE, et al. Posttraumatic stress disorder and extent of trauma exposure as correlates of medical problems and perceived health among women with childhood abuse. *Women and Health* 2001;34(3):1-17. doi: 10.1016/0090-4295(76)90553-7 [published Online First: 1976/01/01]

24. Subica AM, Claypoole KH, Wylie AM. PTSD'S mediation of the relationships between trauma, depression, substance abuse, mental health, and physical health in individuals with severe mental illness: evaluating a comprehensive model. *Schizophrenia research* 2012;136(1-3):104-9. doi: 10.1016/j.schres.2011.10.018 [published Online First: 2011/11/23]

25. National Institute for Clinical Excellence. Post-traumatic stress disorder (PTSD): the treatment of PTSD in adults and children. London: NICE 2005.

26. Bisson JI, Ehlers A, Matthews R, et al. Psychological treatments for chronic post-traumatic stress disorder. Systematic review and meta-analysis. *The British journal of psychiatry : the journal of mental science* 2007;190:97-104. doi: 10.1192/bjp.bp.106.021402 [published Online First: 2007/02/03]

27. Powers MB, Halpern JM, Ferenschak MP, et al. A meta-analytic review of prolonged exposure for posttraumatic stress disorder. *Clin Psychol Rev* 2010;30(6):635-41. doi: 10.1016/j.cpr.2010.04.007 [published Online First: 2010/06/16]

28. Hoppen TH, Morina N. Is high-quality of trials associated with lower treatment efficacy? A meta-analysis on the association between study quality and effect sizes of psychological interventions for pediatric PTSD. *Clin Psychol Rev* 2020;78:101855. doi: 10.1016/j.cpr.2020.101855 [published Online First: 2020/05/04]

For peer review only - http://bmjopen.bmj.com/site/about/guidelines.xhtml

29. Ehlers A, Clark DM. A cognitive model of posttraumatic stress disorder. *Behaviour Research and Therapy* 2000;38(4):319-45. doi: 10.1016/s0300-9629(76)80003-5 [published Online First: 1976/01/01]

30. Ehlers A, Clark DM, Hackmann A, et al. A randomized controlled trial of cognitive therapy, a self-help booklet, and repeated assessments as early interventions for posttraumatic stress disorder. *Arch Gen Psychiat* 2003;60(10):1024-32.

31. Ehlers A, Hackmann A, Grey N, et al. A randomized controlled trial of 7-day intensive and standard weekly cognitive therapy for PTSD and emotion-focused supportive therapy. *Am J Psychiatry* 2014;171(3):294-304. doi: 10.1176/appi.ajp.2013.13040552 [published Online First: 2014/02/01]

32. Meiser-Stedman R, McKinnon A, Dixon C, et al. A core role for cognitive processes in the acute onset and maintenance of post-traumatic stress in children and adolescents. *J Child Psychol Psychiatry* 2019;60(8):875-84. doi: 10.1111/jcpp.13054 [published Online First: 2019/03/27]

33. Smith P, Perrin S, Yule W, et al. Post traumatic stress disorder: Cognitive therapy with children and young people. London: Routledge 2009.

34. Meiser-Stedman R, Smith P, McKinnon A, et al. Cognitive therapy as an early treatment for post-traumatic stress disorder in children and adolescents: a randomized controlled trial addressing preliminary efficacy and mechanisms of action. *J Child Psychol Psychiatry* 2017;58(5):623-33. doi: 10.1111/jcpp.12673 [published Online First: 2016/12/16]

35. Smith P, Yule W, Perrin S, et al. Cognitive-behavioral therapy for PTSD in children and adolescents: a preliminary randomized controlled trial. *J Am Acad Child Adolesc Psychiatry* 2007;46(8):1051-61. [published Online First: 1977/01/01]

36. Sachser C, Keller F, Goldbeck L. Complex PTSD as proposed for ICD-11: validation of a new disorder in children and adolescents and their response to Trauma-Focused Cognitive

For peer review only - http://bmjopen.bmj.com/site/about/guidelines.xhtml

Behavioral Therapy. *J Child Psychol Psychiatry* 2017;58(2):160-68. doi: 10.1111/jcpp.12640 [published Online First: 2016/09/30]

37. Chan AW, Tetzlaff JM, Altman DG, et al. SPIRIT 2013 statement: defining standard protocol items for clinical trials. *Ann Intern Med* 2013;158(3):200-7. doi: 10.7326/0003-4819-158-3-201302050-00583 [published Online First: 2013/01/09]

38. Perrin S, Meiser-Stedman R, Smith P. The Children's Revised Impact of Event Scale (CRIES): Validity as a screening instrument for PTSD. *Behavioural and Cognitive Psychotherapy* 2005;33(4):487-98. doi: 10.1021/bi00622a017 [published Online First: 1977/02/08]

39. Foa EB, McLean CP, Capaldi S, et al. Prolonged exposure vs supportive counseling for sexual abuse–related PTSD in adolescent girls: a randomized clinical trial. *JAMA* 2013;310(24):2650-57. doi: 10.1001/jama.2013.282829 [published Online First: 2013/12/26]

40. Sachser C, Berliner, L., Holt, T., Jensen, T., Jungbluth, N., Risch, E., Rosner, R., Goldbeck, L. International development and validation of the Child and Adolescent Trauma Screening Questionnaire (CATS). International Society for Traumatic Stress Studies. Dallas, USA, 2016.

41. Chorpita BF, Moffitt CE, Gray J. Psychometric properties of the Revised Child Anxiety and Depression Scale in a clinical sample. *Behaviour Research and Therapy* 2005;43(3):309-22. doi: http://dx.doi.org/10.1016/j.brat.2004.02.004 [published Online First: 2005/02/01]

42. Costello EJ, Angold A. Scales to Assess Child and Adolescent Depression - Checklists, Screens, and Nets. *J Am Acad Child Adolesc Psychiatry* 1988;27(6):726-37. doi: 10.1097/00004583-198811000-00011 [published Online First: 1988/11/01]

43. Stringaris A, Goodman R, Ferdinando S, et al. The Affective Reactivity Index: a concise irritability scale for clinical and research settings. *J Child Psychol Psychiatry*

For peer review only - http://bmjopen.bmj.com/site/about/guidelines.xhtml

2012;53(11):1109-17. doi: 10.1111/j.1469-7610.2012.02561.x [published Online First: 2012/05/12]

44. Shaffer D, Gould MS, Brasic J, et al. A children's global assessment scale (CGAS). *Arch Gen Psychiatry* 1983;40(11):1228-31. doi: 10.1001/archpsyc.1983.01790100074010 [published Online First: 1983/11/01]

45. Goodman R. Psychometric properties of the strengths and difficulties questionnaire. *J Am Acad Child Adolesc Psychiatry* 2001;40(11):1337-45. doi: 10.1097/00004583-200111000-00015 [published Online First: 2001/11/09]

46. Goodman M, Patil U, Triebwasser J, et al. Parental burden associated with borderline personality disorder in female offspring. *J Pers Disord* 2011;25(1):59-74. doi: 10.1521/pedi.2011.25.1.59 [published Online First: 2011/02/12]

47. Kroenke K, Spitzer RL, Williams JB. The PHQ-9: validity of a brief depression severity measure. *J Gen Intern Med* 2001;16(9):606-13. doi: 10.1046/j.1525-1497.2001.016009606.x [published Online First: 2001/09/15]

48. Spitzer RL, Kroenke K, Williams JBW, et al. A brief measure for assessing generalized anxiety disorder: the GAD-7. *Archives of Internal Medicine* 2006;166(10):1092-97. doi: 10.1001/archinte.166.10.1092 [published Online First: 2006/05/24]

49. Foa EB, McLean CP, Zang Y, et al. Psychometric Properties of the Posttraumatic Diagnostic Scale for DSM-5 (PDS-5). *Psychol Assess* 2015;28(10):1166-71. doi: 10.1037/pas0000258 [published Online First: 2015/12/23]

50. Byford S, Barrett B, Roberts C, et al. Cost-effectiveness of selective serotonin reuptake inhibitors and routine specialist care with and without cognitive–behavioural therapy in adolescents with major depression. *The British Journal of Psychiatry* 2007;191(6):521-27.

For peer review only - http://bmjopen.bmj.com/site/about/guidelines.xhtml

51. Wille N, Badia X, Bonsel G, et al. Development of the EQ-5D-Y: a child-friendly version of the EQ-5D. *Quality of life research : an international journal of quality of life aspects of treatment, care and rehabilitation* 2010;19(6):875-86. doi: 10.1007/s11136-010-9648-y [published Online First: 2010/04/21]

52. Anilmis JV, Stewart CS, Roddy S, et al. Understanding the relationship between schematic beliefs, bullying, and unusual experiences in 8-14 year olds. *European psychiatry : the journal of the Association of European Psychiatrists* 2015;30(8):920-3. doi: 10.1016/j.eurpsy.2015.08.008 [published Online First: 2015/12/10]

53. Zimet GD, Dahlem NW, Zimet SG, et al. The Multidimensional Scale of Perceived Social Support. *J Pers Assess* 1988;52(1):30 - 41.

54. Meiser-Stedman R, Smith P, Bryant R, et al. Development and validation of the Child Post-Traumatic Cognitions Inventory (CPTCI). *J Child Psychol Psychiatry* 2009;50(4):432-40. doi: 10.1111/j.1469-7610.2008.01995.x [published Online First: 2009/04/03]

55. Meiser-Stedman R, Smith P, Yule W, et al. The Trauma Memory Quality Questionnaire: preliminary development and validation of a measure of trauma memory characteristics for children and adolescents. *Memory* 2007;15(3):271-9. doi: 10.1080/09658210701256498 [published Online First: 2007/04/25]

56. Meiser-Stedman R, Shepperd A, Glucksman E, et al. Thought control strategies and rumination in youth with acute stress disorder and posttraumatic stress disorder following single-event trauma. *J Child Adolesc Psychopharmacol* 2014;24(1):47-51. doi: 10.1089/cap.2013.0052 [published Online First: 2014/02/14]

57. Alberici A, Meiser-Stedman R, Claxton J, et al. The Preliminary Development and Validation of a Trauma-Related Safety-Seeking Behavior Measure for Youth: The Child Safety Behavior

For peer review only - http://bmjopen.bmj.com/site/about/guidelines.xhtml

Scale (CSBS). *J Trauma Stress* 2018;31(5):643-53. doi: 10.1002/jts.22332 [published Online First: 2018/10/20]

58. Shirk SR, Saiz CC. Clinical, empirical, and developmental perspectives on the therapeutic relationship in child psychotherapy. *Dev Psychopathol* 1992;4(04):713-28.

59. Guest G, Bunce A, Johnson L. How many interviews are enough? An experiment with data saturation and variability. *Field methods* 2006;18(1):59-82.

60. Cary CE, McMillen JC. The data behind the dissemination: a systematic review of trauma-focused cognitive behavioral therapy for use with children and youth. *Children and Youth Services Review* 2012;34(4):748-57.

61. Gutermann J, Schreiber F, Matulis S, et al. Psychological Treatments for Symptoms of Posttraumatic Stress Disorder in Children, Adolescents, and Young Adults: A Meta-Analysis. *Clin Child Fam Psychol Rev* 2016;19(2):77-93. doi: 10.1007/s10567-016-0202-5 [published Online First: 2016/04/10]

62. Kraemer HC, Wilson GT, Fairburn CG, et al. Mediators and moderators of treatment effects in randomized clinical trials. *Arch Gen Psychiatry* 2002;59(10):877-83. doi: 10.1001/archpsyc.59.10.877 [published Online First: 2002/10/09]

63. Braun V, Clarke V. Using thematic analysis in psychology. *Qualitative research in psychology* 2006;3(2):77-101.

64. Barry CA, Britten N, Barber N, et al. Using reflexivity to optimize teamwork in qualitative research. *Qualitative health research* 1999;9(1):26-44.

65. National Institute for Health and Care Excellence. Guide to the methods of technology appraisal. London: NICE 2013.

66. Drummond MF, Sculpher MJ, Claxton K, et al. Methods for the economic evaluation of health care programmes: Oxford university press 2015.

For peer review only - http://bmjopen.bmj.com/site/about/guidelines.xhtml

67. Thompson SG, Barber JA. How should cost data in pragmatic randomised trials be analysed? *Bmj* 2000;320(7243):1197-200.

68. Manca A, Hawkins N, Sculpher MJ. Estimating mean QALYs in trial-based cost-effectiveness analysis: the importance of controlling for baseline utility. *Health economics* 2005;14(5):487-96. doi: 10.1002/hec.944 [published Online First: 2004/10/22]

69. Briggs AH. A Bayesian approach to stochastic cost-effectiveness analysis. *Health economics* 1999;8(3):257-61. doi: 10.1002/(sici)1099-1050(199905)8:3<257::aid-hec427>3.0.co;2-e [published Online First: 1999/05/29]

70. Fenwick E, Byford S. A guide to cost-effectiveness acceptability curves. *The British Journal of Psychiatry* 2005;187(2):106-08. doi: 10.1192/bjp.187.2.106 [published Online First: 2005/08/02]

71. Assmann SF, Pocock SJ, Enos LE, et al. Subgroup analysis and other (mis) uses of baseline data in clinical trials. *The Lancet* 2000;355(9209):1064-69. doi: 10.1016/S0140-6736(00)02039-0 [published Online First: 2000/04/01]

For peer review only

For peer review only - http://bmjopen.bmj.com/site/about/guidelines.xhtml

Figure 1. Standard Protocol Items: Recommendations for Interventional Trials (SPIRIT) diagram detailing trial activities and measures and their timing.

Figure 2. Trial Consolidated Standards of Reporting Trials (CONSORT) diagram

For peer review only

For peer review only - http://bmjopen.bmj.com/site/about/guidelines.xhtml

		STUDY PERIOD					
	Screening	Baseline	Allocation	Post-allocation			
TIMEPOINT	$-t_1$	$-t_2$	0	*Mid [2.5m]*	*Post [5m]*	*Follow up [11m]*	*Follow up [29m]*
ENROLMENT:							
Eligibility screen	X						
Informed consent		X					
PTSD severity (CRIES-8)		X		X	X	X	X
PTSD diagnosis (CPSS-I-5)		X			X	X	X
Allocation			X				
INTERVENTIONS:							
CT-PTSD			←	—	→		
TAU			←	—	→		
ASSESSMENTS:							
Demographics & life history		X					
PTSD severity, DSM-5 (CATS)		X		X	X	X	X
Anxiety & depression (RCADS)		X			X	X	X
Suicidal ideation (MFQ)		X			X		
Affect regulation (ARI)		X			X	X	
Functioning (CGAS)		X			X	X	X
Parent/caregiver-report SDQ		X			X	X	X
Borderline traits (MSI-BPD-C)		X			X	X	
Caregiver depression (PHQ-9)		X			X	X	
Caregiver anxiety (GAD-7)		X			X	X	
Caregiver post-traumatic stress (PDS-5)		X			X	X	
Health & social care resource use (CA-SUS)		X			X	X	
Health-related quality of life (EQ-5D-Y)		X			X	X	
Voices (interview)		X			X		
Trauma-related appraisals (CPTCI)		X		X	X		
Trauma memory quality (TMQQ)		X		X	X		
Rumination & self-blame		X		X	X		
Safety-seeking behaviours (CSBS)		X		X	X		
Social support (MSPSS)		X		X	X		
Treatment credibility scale				X	X		
Therapeutic alliance (TASC-r)				X	X		
Adverse events		X			X	X	

Figure 1. Standard Protocol Items: Recommendations for Interventional Trials (SPIRIT) diagram detailing trial activities and measures and their timing.

259x334mm (300 x 300 DPI)

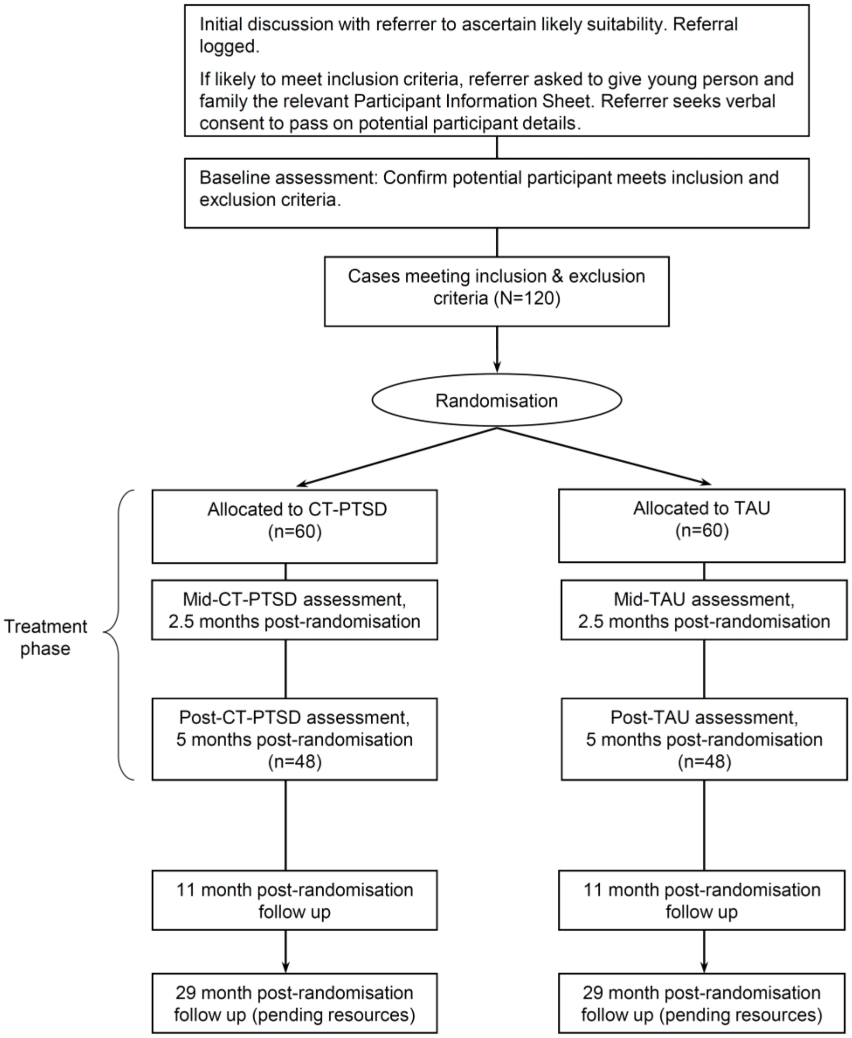

Figure 2. Trial Consolidated Standards of Reporting Trials (CONSORT) diagram

380x400mm (300 x 300 DPI)

SPIRIT
STANDARD PROTOCOL ITEMS: RECOMMENDATIONS FOR INTERVENTIONAL TRIALS

SPIRIT 2013 Checklist: Recommended items to address in a clinical trial protocol and related documents*

Section/item	ItemNo	Description	Page
**Administrative information**			
Title	1	Descriptive title identifying the study design, population, interventions, and, if applicable, trial acronym	1
Trial registration	2a	Trial identifier and registry name. If not yet registered, name of intended registry	3
	2b	All items from the World Health Organization Trial Registration Data Set	4
Protocol version	3	Date and version identifier	4
Funding	4	Sources and types of financial, material, and other support	23
Roles and responsibilities	5a	Names, affiliations, and roles of protocol contributors	23
	5b	Name and contact information for the trial sponsor	23
	5c	Role of study sponsor and funders, if any, in study design; collection, management, analysis, and interpretation of data; writing of the report; and the decision to submit the report for publication, including whether they will have ultimate authority over any of these activities	23
	5d	Composition, roles, and responsibilities of the coordinating centre, steering committee, endpoint adjudication committee, data management team, and other individuals or groups overseeing the trial, if applicable (see Item 21a for data monitoring committee)	17, 19, 20, 24
**Introduction**			
Background and rationale	6a	Description of research question and justification for undertaking the trial, including summary of relevant studies (published and unpublished) examining benefits and harms for each intervention	5
	6b	Explanation for choice of comparators	5-8

For peer review only - http://bmjopen.bmj.com/site/about/guidelines.xhtml

Objectives	7	Specific objectives or hypotheses	8
Trial design	8	Description of trial design including type of trial (eg, parallel group, crossover, factorial, single group), allocation ratio, and framework (eg, superiority, equivalence, noninferiority, exploratory)	8-9
**Methods: Participants, interventions, and outcomes**			
Study setting	9	Description of study settings (eg, community clinic, academic hospital) and list of countries where data will be collected. Reference to where list of study sites can be obtained	9
Eligibility criteria	10	Inclusion and exclusion criteria for participants. If applicable, eligibility criteria for study centres and individuals who will perform the interventions (eg, surgeons, psychotherapists)	9
Interventions	11a	Interventions for each group with sufficient detail to allow replication, including how and when they will be administered	10-11
	11b	Criteria for discontinuing or modifying allocated interventions for a given trial participant (eg, drug dose change in response to harms, participant request, or improving/worsening disease)	n/a
	11c	Strategies to improve adherence to intervention protocols, and any procedures for monitoring adherence (eg, drug tablet return, laboratory tests)	11
	11d	Relevant concomitant care and interventions that are permitted or prohibited during the trial	11
Outcomes	12	Primary, secondary, and other outcomes, including the specific measurement variable (eg, systolic blood pressure), analysis metric (eg, change from baseline, final value, time to event), method of aggregation (eg, median, proportion), and time point for each outcome. Explanation of the clinical relevance of chosen efficacy and harm outcomes is strongly recommended	11-14
Participant timeline	13	Time schedule of enrolment, interventions (including any run-ins and washouts), assessments, and visits for participants. A schematic diagram is highly recommended (see Figure)	14; Fig 1 (p35)

Sample size	14	Estimated number of participants needed to achieve study objectives and how it was determined, including clinical and statistical assumptions supporting any sample size calculations	14
Recruitment	15	Strategies for achieving adequate participant enrolment to reach target sample size	15
**Methods: Assignment of interventions (for controlled trials)**			
Allocation:			
Sequence generation	16a	Method of generating the allocation sequence (eg, computer-generated random numbers), and list of any factors for stratification. To reduce predictability of a random sequence, details of any planned restriction (eg, blocking) should be provided in a separate document that is unavailable to those who enrol participants or assign interventions	16
Allocation concealment mechanism	16b	Mechanism of implementing the allocation sequence (eg, central telephone; sequentially numbered, opaque, sealed envelopes), describing any steps to conceal the sequence until interventions are assigned	16
Implementation	16c	Who will generate the allocation sequence, who will enrol participants, and who will assign participants to interventions	16
Blinding (masking)	17a	Who will be blinded after assignment to interventions (eg, trial participants, care providers, outcome assessors, data analysts), and how	16
	17b	If blinded, circumstances under which unblinding is permissible, and procedure for revealing a participant's allocated intervention during the trial	n/a
**Methods: Data collection, management, and analysis**			
Data collection methods	18a	Plans for assessment and collection of outcome, baseline, and other trial data, including any related processes to promote data quality (eg, duplicate measurements, training of assessors) and a description of study instruments (eg, questionnaires, laboratory tests) along with their reliability and validity, if known. Reference to where data collection forms can be found, if not in the protocol	16*17
	18b	Plans to promote participant retention and complete follow-up, including list of any outcome data to be collected for participants who discontinue or deviate from intervention protocols	n/a

For peer review only - http://bmjopen.bmj.com/site/about/guidelines.xhtml

Data management	19	Plans for data entry, coding, security, and storage, including any related processes to promote data quality (eg, double data entry; range checks for data values). Reference to where details of data management procedures can be found, if not in the protocol	17
Statistical methods	20a	Statistical methods for analysing primary and secondary outcomes. Reference to where other details of the statistical analysis plan can be found, if not in the protocol	17
	20b	Methods for any additional analyses (eg, subgroup and adjusted analyses)	18-19
	20c	Definition of analysis population relating to protocol non-adherence (eg, as randomised analysis), and any statistical methods to handle missing data (eg, multiple imputation)	n/a
**Methods: Monitoring**			
Data monitoring	21a	Composition of data monitoring committee (DMC); summary of its role and reporting structure; statement of whether it is independent from the sponsor and competing interests; and reference to where further details about its charter can be found, if not in the protocol. Alternatively, an explanation of why a DMC is not needed	19-20
	21b	Description of any interim analyses and stopping guidelines, including who will have access to these interim results and make the final decision to terminate the trial	19-20
Harms	22	Plans for collecting, assessing, reporting, and managing solicited and spontaneously reported adverse events and other unintended effects of trial interventions or trial conduct	20
Auditing	23	Frequency and procedures for auditing trial conduct, if any, and whether the process will be independent from investigators and the sponsor	n/a
**Ethics and dissemination**			
Research ethics approval	24	Plans for seeking research ethics committee/institutional review board (REC/IRB) approval	20

For peer review only - http://bmjopen.bmj.com/site/about/guidelines.xhtml

Protocol amendments	25	Plans for communicating important protocol modifications (eg, changes to eligibility criteria, outcomes, analyses) to relevant parties (eg, investigators, REC/IRBs, trial participants, trial registries, journals, regulators)	20
Consent or assent	26a	Who will obtain informed consent or assent from potential trial participants or authorised surrogates, and how (see Item 32)	21
	26b	Additional consent provisions for collection and use of participant data and biological specimens in ancillary studies, if applicable	21
Confidentiality	27	How personal information about potential and enrolled participants will be collected, shared, and maintained in order to protect confidentiality before, during, and after the trial	21
Declaration of interests	28	Financial and other competing interests for principal investigators for the overall trial and each study site	21
Access to data	29	Statement of who will have access to the final trial dataset, and disclosure of contractual agreements that limit such access for investigators	n/a
Ancillary and post-trial care	30	Provisions, if any, for ancillary and post-trial care, and for compensation to those who suffer harm from trial participation	n/a
Dissemination policy	31a	Plans for investigators and sponsor to communicate trial results to participants, healthcare professionals, the public, and other relevant groups (eg, via publication, reporting in results databases, or other data sharing arrangements), including any publication restrictions	21
	31b	Authorship eligibility guidelines and any intended use of professional writers	n/a
	31c	Plans, if any, for granting public access to the full protocol, participant-level dataset, and statistical code	n/a
**Appendices**			
Informed consent materials	32	Model consent form and other related documentation given to participants and authorised surrogates	n/a
Biological specimens	33	Plans for collection, laboratory evaluation, and storage of biological specimens for genetic or molecular analysis in the current trial and for future use in ancillary studies, if applicable	n/a

For peer review only

*It is strongly recommended that this checklist be read in conjunction with the SPIRIT 2013 Explanation & Elaboration for important clarification on the items. Amendments to the protocol should be tracked and dated. The SPIRIT checklist is copyrighted by the SPIRIT Group under the Creative Commons "Attribution-NonCommercial-NoDerivs 3.0 Unported" license.

For peer review only
